# Survival-LCS: Rule-Based Survival Analysis without Proportional Hazard Assumptions

**DOI:** 10.1145/3772719

**Published:** 2026-06-29

**Authors:** ALEXA A. WOODWARD, HARSH BANDHEY, JASON H. MOORE, RYAN J. URBANOWICZ

**Affiliations:** HealthVerity, Inc., Philadelphia, PA, USA; Cedars-Sinai Medical Center, Los Angeles, CA, USA; Cedars-Sinai Medical Center, Los Angeles, CA, USA; Cedars-Sinai Medical Center, Los Angeles, CA, USA

**Keywords:** Learning Classifier Systems, Rule-based Machine Learning, Survival Analysis, Genetic Algorithm

## Abstract

Survival analysis is widely utilized to model time-to-event data across biomedical, epidemiological, and engineering domains. However, traditional methods such as Cox regression impose strong assumptions, including proportional hazards, and are often inadequate for capturing complex, non-linear relationships in high-dimensional or heterogeneous data. Rule-based machine learning algorithms, such as “ExSTraCS,” can interpretably model complex biomedical associations in classification tasks. This study extends ExSTraCS to the challenges of right-censored survival data to yield “Survival-LCS,” the first rule-based survival analysis algorithm that additionally handles heterogeneous feature types and missing values and makes no assumptions about baseline hazard or survival time distributions.

Survival-LCS is evaluated and compared across a variety of simulated genetic survival datasets covering distinct genetic architectures (additive, epistatic, heterogeneous, and univariate), censoring values, number of features, and survival distributions (random monotonic spline, Gamma, Gaussian, and Weibull) using Integrated Brier Scores and statistical significance testing. Results demonstrate that Survival-LCS reliably captures complex associations with survival outcomes, performing competitively with standard approaches, particularly in settings that challenge traditional models. This work highlights the capability of rule-based algorithms to be adapted as interpretable, assumption-free (i.e., data-driven) survival analysis methods, particularly in domains with higher-dimensional data or complex underlying associations.

## 1 Introduction

Survival analysis is a fundamental statistical approach for modeling the time until an event of interest occurs, such as death, disease recurrence, or equipment failure. It is widely applied in biomedical research and epidemiology, as well as in engineering and econometrics [Momtaz, 2021; Zhang et al., 2014]. A key challenge in survival analysis is handling censored data, where the exact time of the event is unknown for some instances (e.g., patients). Specifically, *right-censoring* occurs when we know that an event happened after a certain time point, but the event itself was not observed. This can happen when a study concludes before the event has occurred, for an instance, or if the instance becomes unobservable past some time point in the study. Effectively handling such censored instances is crucial for both traditional parametric and modern machine-learning survival analysis methods [Baek et al., 2021; Binder and Schumacher, 2008; Cox, 1972; Ishwaran et al., 2008; Zhang and Lu, 2007].

One of the most popular methods for survival analysis is the **Cox proportional hazards (CPH)** model. This model includes an unspecified baseline hazard and uses the concept of partial likelihood, which allows it to work with datasets that have many independent variables. Enhancements to the CPH model, such as regularization [Tibshirani, 1997; Zhang and Lu, 2007] and boosting [Binder and Schumacher, 2008], have improved its performance. However, the CPH model and its extensions rely on the **proportional hazards (PH)** assumption. This assumption posits that the hazard, or the rate at which events occur, is proportional across different values of a predictor variable over time. This assumption, and having an unspecified baseline hazard, adds another layer of difficulty, since a survival distribution needs to be specified in order to make predictions. Furthermore, like many other regression models, the CPH model assumes linearity and additivity of predictor variables [Kuitunen et al., 2021]. These assumptions often do not hold in real-world scenarios, which can limit the effectiveness of the model.

To address these limitations, machine learning techniques have also been introduced for survival analysis. These include support vector machines [Sun et al., 2020; Van Belle et al., 2007], **random survival forests (RSFs)** [Ishwaran et al., 2008; Morsy et al., 2021], and deep learning methods [Baek et al., 2021; Mobadersany et al., 2018; Salimy et al., 2023]. While these models can improve predictive performance, they have other limitations. For example, RSFs often use the log-rank statistic for data splitting, which, like the PH assumption, can impose certain constraints on the data [Nasejje et al., 2017]. Deep learning methods, while powerful, typically require large sample sizes for training [Yousefi et al., 2017] and often lack interpretability. Also of note, many popular survival analysis techniques struggle with detecting complex associations, such as gene–gene interactions (epistasis) [Urbanowicz et al., 2012d] and heterogeneous effects across subpopulations [Woodward et al., 2022], particularly in high-dimensional data with more variables than samples. This can lead to overfitting and reduced generalizability [Zhao and Li, 2012]. These challenges highlight the need for survival models that are assumption-free, interpretable, and capable of uncovering complex, non-linear relationships in heterogeneous and high-dimensional data. Toward this end, we propose *Survival-LCS*, a novel **learning classifier system (LCS)** designed for survival analysis.

*LCSs* are rule-based machine learning algorithms that use evolutionary search to discover a population of human-interpretable IF:THEN rules that together serve as a predictive model. LCSs are well suited for modeling complex, noisy, or heterogeneous data due to their flexible structure and capacity for global and local pattern discovery [Urbanowicz and Browne, 2017; Urbanowicz and Moore, 2009]. Despite their success in classification and regression tasks, LCSs have not previously been adapted for survival analysis prior to our proposed method [Woodward et al., 2024]. Survival-LCS is based on ExSTraCS, an LCS originally designed for biomedical classification tasks [Urbanowicz and Moore, 2015]. ExSTraCS has shown effectiveness in modeling complex multivariate patterns in both clean and noisy-signal classification problems [Urbanowicz and Moore, 2015; Urbanowicz et al., 2015, 2016]. We extend this framework to accommodate survival data by introducing novel mechanisms tailored to quantitative outcomes and right-censoring. Specifically, we define the concept of an event *interval*, adapt the prediction and error update mechanisms to handle censoring, and incorporate a previously proposed multi-objective optimization strategy [Urbanowicz et al., 2016] to balance rule accuracy and generalization. The proposed Survival-LCS system offers multiple advantages: (1) It requires no assumptions about the baseline hazard or survival time distribution, making it suitable for non-PH scenarios. (2) It generates interpretable models composed of rule sets, facilitating transparency and explainability. (3) It is capable of modeling complex multivariate associations. (4) It supports missing data, diverse feature types, and larger feature sets, making it well suited for biomedical applications where these characteristics are common.

This study builds upon the original work by Woodward et al. [2024], extending the previous analyses to explore the capabilities and limitations of Survival-LCS via (1) using broader hyperparameter resource settings, i.e., number of training iterations and population size and (2) examining a variety of survival distributions, i.e., directly testing the ability of Survival-LCS to perform in datasets without relying on the PH assumption. By systematically assessing the performance of Survival-LCS across different genetic architectures, censoring proportions, **minor allele frequencies (MAF)**, and survival distributions, we aim to uncover critical insights into the algorithm’s robustness, versatility, and areas for improvement. These findings contribute to a deeper understanding of Survival-LCS’s potential for addressing challenges in survival analysis and its applicability in complex real-world scenarios. The subsequent sections of this paper provide an overview of the Survival-LCS methodology, the strategy used to evaluate and compare Survival-LCS across a variety of simulated right-censored survival datasets, results, discussion, and conclusions.

## 2 Methods

This section details the Survival-LCS algorithm as well as the experimental evaluation utilized in this study.

### 2.1 Survival-LCS Algorithm

The Survival-LCS algorithm is an adaptation of LCSs for survival analysis tasks [Woodward et al., 2024]. LCSs are evolutionary rule-based machine learning algorithms that train a population of IF:THEN rule expressions that collectively form a model to tackle reinforcement or supervised learning tasks [Urbanowicz and Browne, 2017]. We begin by outlining the algorithm lineage, then explain the basics of how LCS algorithms work, and finally detail differences between Survival-LCS and other LCSs algorithms that allow adaptation to survival analyses.

#### 2.1.1 Algorithm Lineage.

Survival-LCS is most closely based on the ExSTraCS 2.0 algorithm [Urbanowicz and Moore, 2015], previously designed for scalable biomedical classification tasks. ExSTraCS 2.0 is descended from a lineage of Michigan-style LCSs including XCS [Wilson, 1995] the most popular LCS to date; UCS [Bernadó-Mansilla and Garrell-Guiu, 2003] a specialization of XCS for supervised learning tasks, and ExSTraCS 1.0 [Urbanowicz et al., 2014] an adaptation of UCS for noisy biomedical data analyses.

ExSTraCS 1.0 extended the UCS framework with a variety of novel heuristics aimed at improving performance and model interpretability in noisy, larger-scale classification datasets, including attribute tracking/feedback, expert knowledge covering, a flexible rule representation strategy, and a rapid rule compaction strategy [Urbanowicz et al., 2014]. ExSTraCS 2.0 further added strategies to improve algorithm scalability to increasing dataset sizes and was one of the first algorithms to successfully tackle the extremely complex 135-bit multiplexer benchmark problem [Urbanowicz and Moore, 2015]. Solving this problem requires the identification of 128 heterogeneous eight-way epistatic interactions, which highlights the power of LCS algorithms to capture extremely complex relationships in data.

**Table:** 

Algorithm 1: Survival-LCS Algorithm Pseudo-Code
$$\begin{array}{cc} \; & \;1:\text{Initialize empty}\;\mathrm{rule}\;\mathrm{population}\;[P]\\ \; & \;2:\mathrm{for}\;\text{each training iteration in}\;max\_iterations\;\text{get the next training instance}\;x\;\mathrm{do}\\ \; & \;3:\;\;\;\text{Form}\;\mathrm{match}\;\mathrm{set}\;[M]\;\text{from}\;[P]\;\text{for instance}\;x\\ \; & \;4:\;\;\;\mathrm{if}\;\text{no existing rules match}\;x\;\mathrm{then}\\ \; & \;5:\;\;\;\;\;\text{Create new rule using}\;\mathrm{covering}\;\text{and add to}\;[P]\\ \; & \;6:\;\;\;\mathrm{end}\;\mathrm{if}\\ \; & \;7:\;\;\;\text{Partition}\;[M]\;\text{into}\;\mathrm{correct}\;\mathrm{set}\;[C]\;\text{and}\;\mathrm{incorrect}\;\mathrm{set}\;[I]\;\text{based on outcome alignment}\\ \; & \;8:\;\;\;\text{Update}\;\mathrm{rule}\;\mathrm{parameters}\;(\text{e.g.}\;\text{accuracy},\;\text{fitness},\;\text{etc}.)\;‘\text{batch-wise}’\\ \; & \;9:\;\;\;\text{Apply}\;\mathrm{subsumption}\;\text{to generalize overfit rules}\\ \; & \;10:\;\;\;\text{Apply}\;\mathrm{genetic}\;\mathrm{algorithm}\\ \; & \;11:\;\;\;\text{Select parents from}\;[C]\;\text{using}\;\mathrm{tournament}\;\mathrm{selection}\\ \; & \;12:\;\;\;\text{Apply crossover and mutation to generate new offspring rules}\\ \; & \;13:\;\;\;\text{Insert new offspring into population}\;[P]\\ \; & \;14:\;\;\;\text{Apply}\;\mathrm{probabilistic}\;\mathrm{deletion}\;\text{to maintain}\;max\_pop\_size\\ \; & \;15:\mathrm{end}\;\mathrm{for} \end{array}$$

Survival-LCS also incorporates algorithmic elements proposed to adapt ExSTraCS 2.0 to quantitative outcome predictions [Urbanowicz et al., 2015] and a multi-objective optimization strategy for evaluating discovered rules to further improve performance in noisy problem domains [Urbanowicz et al., 2016].

#### 2.1.2. Basic LCS Algorithm Overview.

This section reviews the core components of the Survival-LCS algorithm that are identical to the ExSTraCS 2.0 algorithm and very similar to other Michigan-style LCS algorithms, including UCS and XCS.

First, it’s important to understand that LCS rules take the IF:THEN form (i.e., condition:action) mapping one or more feature states to a predicted outcome, e.g., IF “*Gene-A*” is state 0 and “*body-mass-index*” has a state between 20 and 30, THEN predict “healthy.” Survival-LCS rules are similar; however, the *action* of rules is replaced with a predicted *event interval* rather than a classification prediction, as described later. Each LCS rule also maintains a number of other parameters that capture other key rule characteristics, including rule accuracy, *numerosity* (i.e., the number of virtual copies of the rule in the rule population), number of training instances matched by rule, rule fitness, etc.

Another key element of the LCS algorithm is the rule population [P]. This is the set of all unique rules that have been discovered and maintained within the algorithm. At the end of algorithm training, [P] constitutes the trained LCS model that can be used to make predictions with the help of the *prediction array*. Two critical hyperparameters for LCS include the maximum rule population size, *max_pop_size* and the maximum number of training iterations, *max_iterations*.

**Fig. 1.**
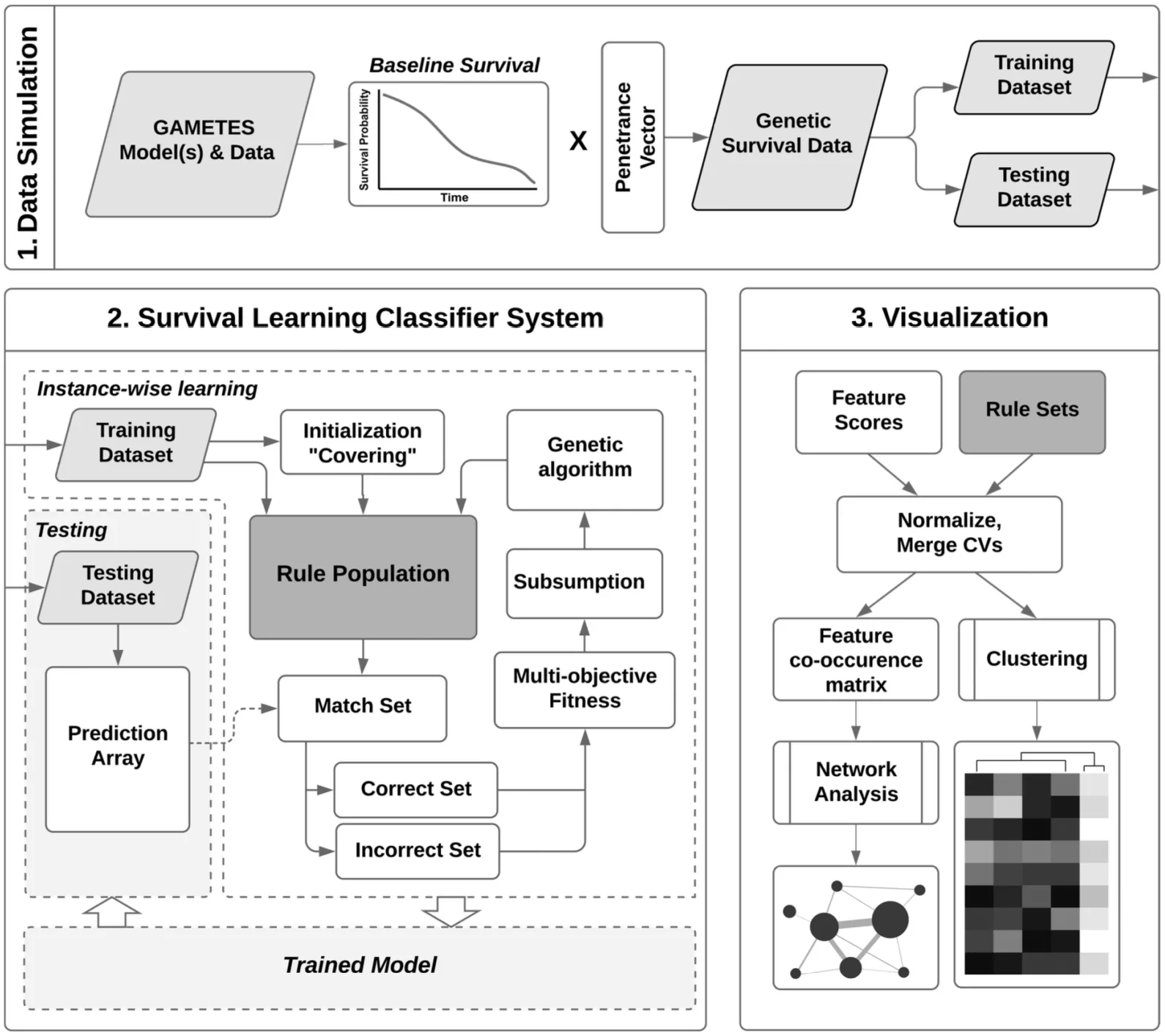
The experimental study design of Survival-LCS consists of three stages: (1) Data Simulation uses GAMETES to generate genetic models with survival outcomes by applying a baseline survival curve to penetrance vectors. (2) Survival-LCS incrementally trains a rule population over the course of its evolutionary training iterations. The trained model consists of a population of individually interpretable IF:THEN rules that each predict survival intervals. (3) Visualization via the LCS-Dive tool [Zhang et al., 2021] aggregates rule sets and attribute tracking scores across **cross-validation (CV)** folds, enabling clustering and network-based global visual model interpretations. GAMETES, Genetic Architecture Model Emulator for Testing and Evaluating Software.

To summarize how Survival-LCS trains a rule population, an algorithmic description is given in Algorithm 1. Additionally, a visual schematic of the major elements of the Survival-LCS algorithm is also given in Box 2 of Figure 1. The algorithm begins by initializing an empty rule population. Over the course of subsequent training iterations, the algorithm will focus on one “target” training instance (x) at a time, cycling repeatedly through the training dataset until the *max_iterations* are exhausted. A single training iteration consists of multiple steps, as follows. First a *match set*, [M], is formed as the subset of rules from [P] where the state(s) of the “target” instance match the rule’s *condition*. The values of any dataset features that are unspecified in a rule are ignored during matching. If, after matching, [M] is empty (as it certainly would be at the algorithm’s start), then the *covering* operator randomly generates a rule that matches the “target” instance feature states. Of note, Survial-LCS uses the same rule condition representation as ExSTraCS 2.0 and adopts the *rule-specificity limit* strategy, which conservatively limits the number of feature states that can be specified within a given rule based on characteristics of the training dataset [Urbanowicz and Moore, 2015]. Next, [M] is further partitioned into a *correct set* [C], and *incorrect set* [I], where rules in [C] traditionally have the same action as the “target” instance, and those in [I] do not. The definition of [C] and [I] in Survival-LCS has been adapted for survival analysis as described later. Next, key rule parameters such as rule accuracy and fitness are updated. Of note, for most previous LCS algorithms, these updates are incremental, reflecting the new information gained by observing the next training instance in the dataset queue. However, one key difference introduced in Survival-LCS, is that newly introduced rules are evaluated once across all available training instances (i.e., batch-wise evaluation), rather than incrementally over the course of learning iterations. Next, Survival-LCS applies *subsumption*, which serves as an explicit generalization mechanism where simpler rules “consume” more complex rules that cover the same problem subspace but with no greater accuracy. This helps to reduce overfitting. Next, a fairly standard *genetic algorithm* is employed, which probabilistically selects two “parent” rules from [C] using *tournament selection*, mates them using *crossover* and *mutation* operators to generate two “offspring” rules, and inserts any newly discovered rules into [P]. Lastly, the training iteration ends by applying probabilistic *roulette-wheel deletion* to maintain the maximum rule population size. This prevents algorithm memory and runtime bloat. Once trained, the LCS model can be applied to make predictions (e.g., on testing data) using the *prediction array*. The prediction array takes the testing instance, forms an [M] and then identifies the prediction with the most support based on the rules in [M]. For a more in-depth explanation of LCS algorithms or the ExSTraCS 2.0 algorithm (from which Surival-LCS inherits most of its heuristics) we refer readers to Urbanowicz and Browne [2017] and Urbanowicz and Moore [2015], respectively.

#### 2.1.3 General Adaptations to Survival Analyses.

There were three main challenges in adapting ExSTraCS 2.0 to survival analyses: (1) adapting rule prediction from classification to quantitative prediction, i.e., time-to-event, (2) avoiding overfitting and encouraging rule generalizability, and (3) accommodating the uncertainty introduced by censoring. Right censoring is especially common in biomedical studies due to limited study durations, patient dropout, or logistical failures in patient follow-up. Improperly handling censoring, e.g., by naively treating censored instances as complete observations, can bias survival estimates and downstream predictive model performance. Survival-LCS introduces a number of algorithmic modifications to ExSTraCS 2.0 to address these challenges.

First, as previously mentioned, newly evolved rules are evaluated in batch on all available training instances (similar to Pittsburgh-style LCSs [Urbanowicz and Moore, 2009]) rather than incrementally over learning iterations. While incremental rule evaluation is necessary for LCS reinforcement learning (e.g., see XCS [Wilson, 1995]), batch rule evaluation is well-suited to supervised learning tasks and allows simplifications to other aspects of Survival-LCS including the multi-objective fitness function discussed later. Of note, unlike in Pittsburgh-style LCSs, in Survival-LCS, the genetic algorithm still operates at the individual rule level.

A second modification is the use of an “event-interval” as the predicted *action* of a rule, e.g., [*min_time, max_time*]. This is closely inspired by previous work, which implemented a supervised LCS approach to quantitative prediction using “endpoint-intervals,” but did not account for censoring Urbanowicz et al. [2015]. Of note, most LCSs define rule niches (i.e., area of specialization) based on the *condition* component of the rule, which controls which rules match a given instance. While some LCS variants [e.g., Iqbal et al., 2013; Lanzi, 2025] can define niches based on a “computed” action of the rule, which can help to avoid overly specific rule conditions, Survival-LCS relies on traditional condition-to-action mapping in rules in order to prioritize rule interpretability, allowing clinicians or other researchers to be able to clearly trace predictions back to the underlying rule mappings. This is likely to be especially valuable in high-stakes biomedical domains. However, as a consequence, rules with event intervals that are too narrow will rarely make it into correct sets (reducing rule accuracy), and rules with event intervals that are too wide will be overly general and yield less predictive precision of the time-to-event. Survival-LCS seeks to address this by using a multi-objective rule-fitness function that encourages either accuracy and/or correct instance coverage. This is designed to encourage rules to emerge that can identify useful event interval ranges for the target problem.

**Fig. 2.**
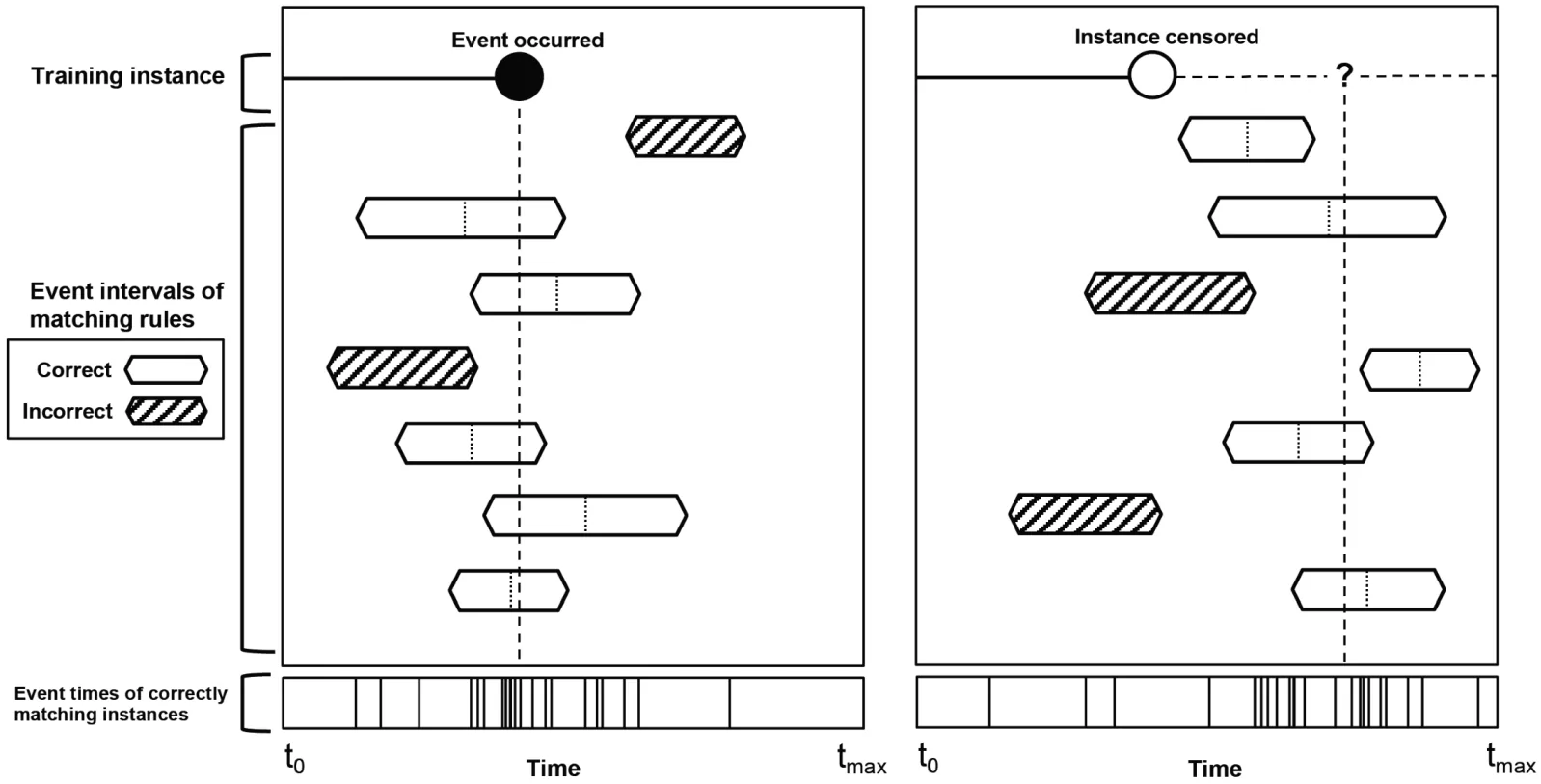
Illustration of Rule Set Formation ([C] and [I]) and Event Interval Evaluation in Survival-LCS: This figure illustrates event intervals for rules that match a given data instance and demonstrates how the Survival-LCS algorithm separates correct from incorrect rules for instances that are either uncensored (left) or right-censored (right). Each hexagonal bar represents the event interval predicted by a matching rule. White bars indicate correct event interval predictions, and diagonally shaded bars indicate incorrect ones. For uncensored instances (left), the event occurs at a known time (black circle), and a rule is considered correct if its predicted interval contains this time. For censored instances (right), where the event has not yet occurred (open circle), rules are correct if their intervals occur after the censoring time, respecting the uncertainty of when the event may occur. The long vertical dotted lines indicate where the true event time for either instance occurred (which wouldn’t be known for censored instances). With respect to making predictions, the dotted vertical lines within individual bars represent the event time predicted for the respective rule. Below each (left/right) panel, we visualize the event times of matching instances from the training dataset for rules in [C]. The density of these times informs the predicted survival distribution.

A third modification involves how [C] or [I] are formed for uncensored vs. censored instances after the formation of [M]. As illustrated in Figure 2, for uncensored instances (i.e., when the event time was observed), rules that have an event interval that contains the instance’s observed event time are placed in [C] (i.e., the event interval “correctly” captured the true event time), and all others are placed in [I]. For censored instances, rules with an event interval that lies entirely after the censored time are placed in [C] (i.e., these rules at least correctly capture that the event happened sometime after the censored time of the instance). Differently, any rule with an interval lower bound that falls before the censored instance time is placed in [I], (i.e., at least part of the predicted time interval incorrectly fell before the censored time). This strategy allows the model to learn from censored data without making strong assumptions about unobserved event times.

The next two subsections outline modifications to Survival-LCS that deal with rule parameter metrics for evaluating individual rules, followed by a subsection detailing how Survival-LCS makes predictions.

#### 2.1.4 Calculating Rule Accuracy.

In other supervised LCSs performing classification, such as UCS [Bernadó-Mansilla and Garrell-Guiu, 2003] and ExSTraCS [Urbanowicz and Moore, 2015], rule accuracy is calculated as the number of times a rule has been in [C] divided by the number of times in [M]. To accommodate the quantitative prediction of survival time, rule accuracy in Survival-LCS is further adjusted based on its ability to predict the actual event time accurately. For rules in the correct set [C], rule error for a specific instance is calculated as the absolute difference between the instance event time (evenTime) and the center of the rule’s event interval ($EI_{C}$), divided by the total time range observed in the dataset ($T_{\max}-T_{\text{min}}$). Of note, Figure 2 illustrates $EI_{C}$s as short vertical dotted lines within event interval bars. Rules falling into the incorrect set [I] are assigned the maximum error of 1, unless a rule’s event interval encompasses the instance’s censoring time. In this case, the error is proportional to the range of the interval that is known to be inaccurate (*propIncorrect*), i.e., the range before the censoring time. For example, if an event is censored at time 12 and a matching but incorrect rule has an interval spanning times 10–16, the error would be calculated as 1 × ((12 – 10)/(16 – 10)) = 0.33. The accuracy for each rule is then calculated as 1 minus the sum of these errors divided by the number of matching instances, as seen in Equation (1):

(1) $$\text{Rule Accuracy}=1-\frac{\text{sumErr}}{\text{matchCover}}.$$

An exception to this accuracy update occurs when a rule correctly matches a censored instance. Since it is uncertain whether an event occurred after censoring, it is impossible to assign an error to correctly matching rules for such instances. Consequently, these instances do not contribute to the accuracy update, meaning no error is added to sumErr. This ensures that censored instances provide information only up to the time of censoring. These error update schemes are summarized in Table 1.

#### 2.1.5 Calculating Multi-Objective Rule Fitness.

To accommodate the dual goals of accuracy and generalizability in the presence of noisy survival data, Survival-LCS incorporates a **Pareto-inspired multi-objective rule fitness (PIMORF)** strategy based on the approach proposed for ExSTraCS [Urbanowicz et al., 2016]. This method enables the simultaneous evaluation of rules along multiple axes without relying on predefined objective weights.

Each rule is evaluated on two objectives: (1) rule accuracy, as defined above for event time prediction and (2) useful coverage, which refers to the number of training instances for which the given rule both matched and was included in [C] (i.e., it was correctly covered) beyond random chance (i.e., beyond the total number of training instances in the dataset where the event time falls within the event interval). Since rules with larger event intervals can match more instances by chance, this definition of coverage ensures that rules with large event intervals don’t arbitrarily dominate the rule population.

Further, rather than combining metrics into a scalar, rule fitness is based on the rule’s distance from a non-dominated Pareto front maintained to represent the best metric tradeoffs of all rules discovered thus far by the algorithm. A rule is non-dominated if no other rule in the population improves one objective without making the other worse. The rule fitness is thus computed as the relative Euclidean distance from the origin (0, 0) to the rule’s point, normalized by the distance to the current Pareto front, as shown in Equation (2):

(2) $$\text{Fitness}(r)=\frac{\left\|\vec{r}\right\|}{\left\|\vec{p^{*}}\right\|},\;\;\text{where}\;\vec{r}=\left(A_{r},\frac{C_{r}}{C_{mac}}\right).$$

Here, $A_{r}$, and $C_{r}$, are the accuracy and useful coverage of rule r, and $\vec{p^{*}}$ is the corresponding point on the Pareto front with its own interval accuracy and useful coverage.

Rules on the non-dominated Pareto front receive a maximum fitness score of 1.0. Dominated rules are scaled proportionally based on their distance to the front. This agnostic formulation avoids biasing toward either objective, making it suitable when signal-to-noise properties of the data are unknown [Urbanowicz et al., 2016].

#### 2.1.6 Making Predictions with Survival-LCS.

After training a Survival-LCS model, it is capable of making predictions about event times as well as generating expected survival distributions for new cases. This flexibility enhances its downstream clinical applicability.

To predict a quantitative event time for a new instance, the prediction array of Survival-LCS first forms [M] from [P], similar to the start of a training iteration. Each rule in [M] is associated with an event interval (defined by upper and lower bounds), a fitness value, and a numerosity. The prediction proceeds through the following steps: (1) The lower and upper bound event times proposed by each rule in [M] are recorded; (2) All recorded bounds are combined into a single list and sorted in ascending order; (3) Contiguous time periods are formed by treating each successive pair of event times in the list as defining a segment of the timeline [see Urbanowicz et al., 2015 for details]; (4) For each period, all rules whose intervals fully cover the period are identified. For these rules, the product of fitness and numerosity is computed and summed to yield the period’s total support score; and (5). The period with the highest total score is identified as the most strongly supported region by the match set. The midpoint of this segment is then reported as the predicted event time.

Beyond producing a point estimate, Survival-LCS can also provide individualized survival probability curves. This extended prediction procedure builds on the same [M] described above and proceeds as follows: (1) For each rule in [M], retrieve the set of training instance event times where that rule was also in [C] (referred to here as *cover times*); (2) Aggregate all cover times across [M] into a single collection. Each cover time is associated with the rule that matched it and inherits the corresponding rule’s fitness value; (3) Using kernel density estimation, derive a probability density function (PDF) from these cover times (see the bottom of Figure 2), where the contribution of each event time point is also weighted by rule fitness; (4) The PDF is then integrated to obtain the cumulative density function (CDF); (5) The predicted survival distribution for the test instance is determined by computing 1 – CDF, which reflects the training data’s survival outcomes.

#### 2.1.7 Code Availability.

The Survival-LCS algorithm, along with the code used for the analyses in this study, is publicly available at https://github.com/UrbsLab/survival-LCS/tree/telo.

### 2.2 Experimental Evaluation

This section outlines our experimental framework for evaluating the performance, robustness, and interpretability of Survival-LCS. We conducted extensive benchmarking using simulated datasets that vary in genetic architecture, baseline survival distributions, feature dimensionality, and censoring values. These experiments were designed to validate the algorithm’s utility in modeling complex time-to-event relationships across a diverse range of scenarios, with an emphasis on both predictive accuracy and interpretability.

#### 2.2.1 Overview.

**Fig. 3.**
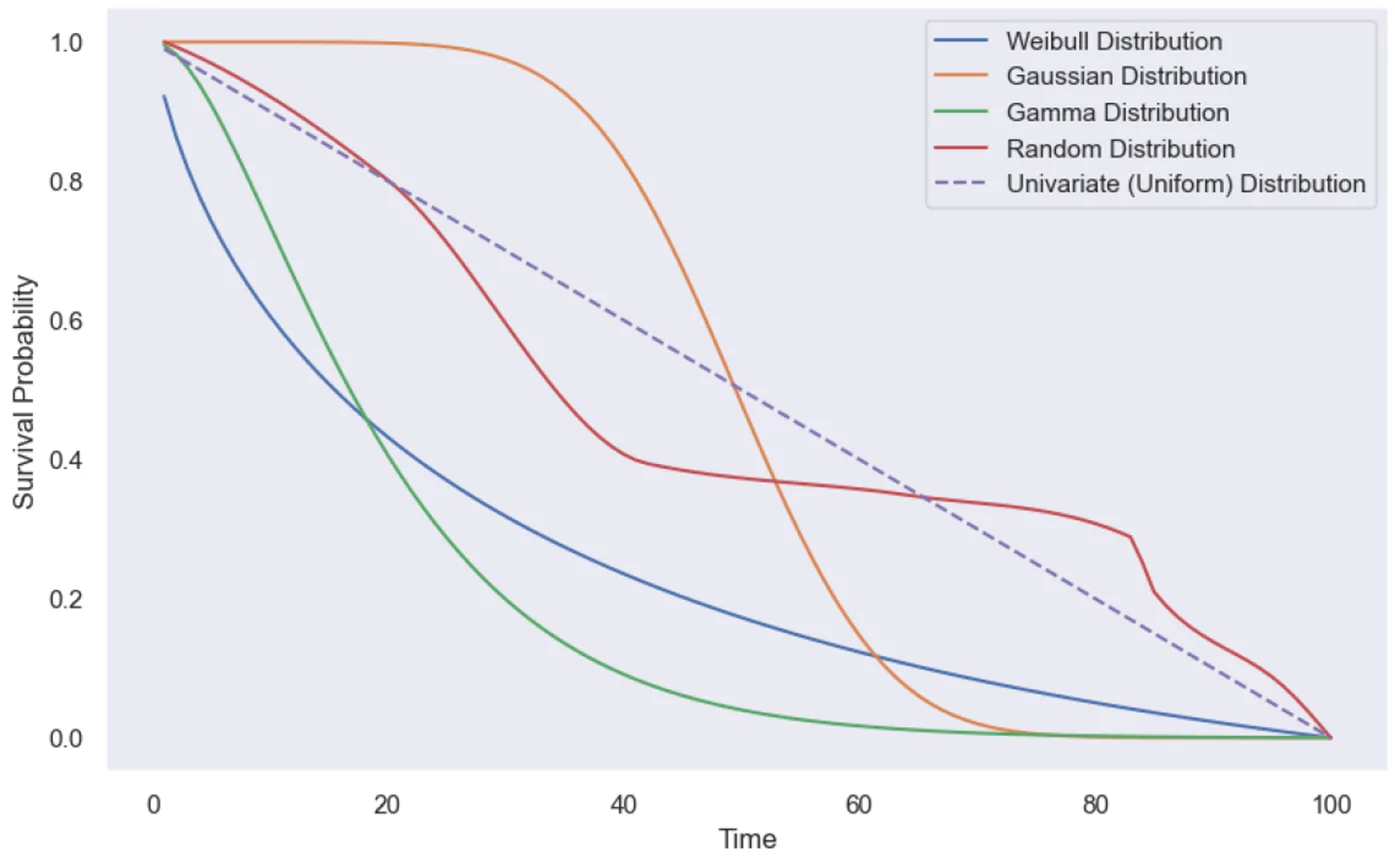
Example survival probability curves across different baseline survival time distributions.

Figure 1 illustrates an overview of the study, including survival data simulation, training predictive models with the Survival-LCS algorithm, model evaluation on testing partitions with an extensive sensitivity analysis, and examinations of model interepretability using the LCS-Dive [Zhang et al., 2021] visualization pipeline. To validate the feasibility of a rule-based approach for survival analysis, we assessed the performance of Survival-LCS across a variety of simulated survival datasets, each exhibiting different patterns of association between genomic features and survival outcomes. The survival data simulations were created by customizing datasets generated with the GAMETES [Urbanowicz et al., 2012d]. GAMETES can produce genetic models and datasets that include simple univariate effects, as well as more complex multivariate additive, epistatic, and heterogeneous associations. In previous work, the GAMETES datasets were only modified to generate survival (time and censoring) outcomes suitable for using a RS distribution (i.e., random distribution) [Woodward et al., 2024]. Here, we extend this simulation study to also consider Gamma (α = 2, γ = 1/10), Gaussian (μ = 0.5, σ = 0.1), and Weibull (κ = 0.63, λ = 0.33) distributions in addition to the random distribution. Figure 3 visualizes example survival probability curves for these four different distributions, in contrast with a simple univariate (uniform) distribution for comparison. Each curve highlights distinct decay patterns over time, emphasizing the diversity in survival trends and the need for flexible models like Survival-LCS to handle varied real-world scenarios.

Simulated survival datasets were generated having a range of dataset parameter configurations as detailed in Table 2 to encompass a broad spectrum (total of 288) of potential real-world dataset characteristics [Urbanowicz et al., 2012d, 2018]. These simulated datasets represent a variety of different baseline distributions, underlying genetic models (with different patterns of association), total numbers of features in the dataset, proportions of censored instances, and MAF of the modeled predictive features. Prior work has shown that lower-fold CV offers comparable variance to higher folds [Kohavi, 1995] when dataset sizes are adequate. We used fivefold CV to balance computational feasibility with reliable performance estimation. Given the exploratory nature of our study and the high computational cost of evolutionary learning algorithms like Survival-LCS, especially across multiple synthetic datasets with varying censoring levels and feature sets, this choice enabled broader evaluation within practical constraints.

#### 2.2.2 Generating Right-Censored Survival Data with GAMETES.

To simulate realistic and diverse associations that map predictive features to a survival time outcome, we used GAMETES to generate genotype-based datasets with known underlying patterns of association [Urbanowicz et al., 2012c,d]. GAMETES allows the creation of additive, epistatic, and heterogeneous genetic models by specifying penetrance functions that map genotype combinations to the probability of expressing one of two possible class outcomes. Table 3 gives an example of a penetrance function generated by GAMETES that specifies a two-way epistatic interaction between two genetic features (each with possible states of 0, 1, or 2). Drawing from Harden and Kropko’s framework [Harden and Kropko, 2019], this study adapted GAMETES to instead generate time-to-event quantitative outcomes along with censoring. For this, outcome probabilities of the penetrance function are instead utilized as a basis for generating similar survival times given a specific genotype combination. For example, the penetrance function in Table 3 can now be interpreted as indicating that when both features have a state of 1, the highest survival times should ultimately be simulated, while when feature 1 has a state of 1 and feature 2 has a state of 2, the lowest survival times should be simulated.

More specifically, the survival data simulation procedure follows the following steps: (1) Generate the baseline hazard function using the method respective to each of the distributions examined in this study (e.g., gamma or random spline); (2) For each data instance, assign an initial survival magnitude by selecting a value from a normal distribution with μ = penetrance value (taken from the GAMETES penetrance function based on the instance’s genotype or genotype combination) and σ = 0.1 (to introduce slight variation and noise); (3) Multiply each survival magnitude by exp(penetrance) at each time point of the baseline hazard, resulting in an N∗t matrix, i.e., a survival function for each subject based on the simulated causal features; (4) Simulate final event times by inverting the CDF: randomly draw a value from U[0,1] and determine the time point at which the survivor function for each instance falls below this random draw, marking it as the event time; (5) Simulate censoring by randomly assigning a proportion of instances a status of 0 (i.e., a censored instance) instead of a status of 1 (i.e., uncensored). For simplicity, censored subjects retain their modeled event time. All survival times in the simulated dataset are generated as whole numbers in the range of [0–100]. Every dataset was simulated to include 1,000 instances. Further details on these simulated datasets are given in Supplementary Material S.1.2.

#### 2.2.3 Sensitivity Analysis.

As mentioned, this study applies an extensive sensitivity analysis to assess the performance and robustness of the Survival-LCS algorithm across a number of simulated survival data scenarios spanning different survival distributions, genetic models, number of features, proportions of censored instances, and MAF. The added exploration of survival distributions beyond the RS provides a broader examination of Survival-LCS’s ability to handle diverse survival patterns, better reflecting real-world possibilities and complexities. For each of the 288 simulated datasets, fivefold CV was employed in the training and evaluation of models.

In this study, Survival-LCS models were trained for 200,000 iterations per CV-fold, with a maximum rule population size of 2,000 in contrast with having used 50,000 iterations and a maximum rule population size of 1,000 in our initial evaluations [Woodward et al., 2024]. As before, all other Survival-LCS hyperparameters were left to default values [Woodward et al., 2024]. All Survival-LCS hyperparameters are documented in Supplementary Material S.1.1 (Supplementary Table S1). This study also employed the **Integrated Brier Score (IBS)** as the key performance metric, which evaluates prediction accuracy across all time points, with lower IBS values indicating superior performance. The IBS was calculated as the trapezoid integration of time-dependent Brier scores at each time point. The individual Brier scores measure the mean squared error between predicted survival probabilities and observed outcomes while accounting for censoring through the inverse probability of censoring weights. To ensure robust evaluation, we restricted predictions and assessments to time points within the range of follow-up times observed in the training data, thereby avoiding issues arising from the inability of various models (such as Cox regression or survival trees) to extrapolate beyond the training time range. For test instances with follow-up times outside this range, predictions were truncated, and the IBS calculation was performed only on the overlapping intervals. Time points for Brier score computation were chosen as evenly spaced intervals within the range of test follow-up times. During CV, test folds contained follow-up times overlapping with the training fold range, as both are generated in the range of [0–100]. These methodological considerations ensure the validity of the IBS as a performance metric for survival models in the presence of censored data. Confidence interval results for IBS were calculated and can be found in the Supplementary Materials to provide a statistical context for model performance.

To further validate the results, we performed permutation testing on datasets with 100 features. For each of the five CV folds, 20 permutations were generated (100 permutations in total), where survival times were randomly shuffled to empirically assess the significance of model predictions. A Wilcoxon-Rank Sum Test was then applied to the resulting Brier scores, providing a benchmark for distinguishing true predictive signal from random noise in high-dimensional settings. We also compared Survival-LCS to the CPH model, implemented using the scikit-survival Python library [Pölsterl, 2020]. As a widely used and interpretable baseline, the standard CPH model served as a reference point for evaluating traditional survival analysis approaches. However, due to convergence issues in high-dimensional scenarios, CPH was applied only to datasets with 100 features (also highlighting a fundamental limitation of these approaches). Permutation testing was similarly limited to these lower-dimensional settings due to computational complexity. As this study is exploratory in nature and aims to uncover broad performance trends across varied survival scenarios, we did not apply Bonferroni or other multiple comparison corrections. Many of the comparisons involve overlapping or correlated datasets (e.g., identical features with different censoring levels), and applying strict correction procedures could increase the risk of Type II errors and obscure meaningful insights.

Finally, we utilized the LCS-DIVE [Zhang et al., 2021] pipeline to analyze selected models, enabling a deeper understanding of Survival-LCS’s ability to differentiate between predictive and non-predictive features. This visualization technique revealed how the algorithm captured the true underlying genetic models, demonstrating its capacity to uncover and characterize complex patterns of association in survival data.

#### 2.2.4 Data Availability.

All simulated datasets used in this study, including the GAMETES model files and corresponding CV datasets, are publicly available to support reproducibility and enable future benchmarking by the research community. They can be accessed at: https://doi.org/10.5281/zenodo.15243723.

## 3 Results and Discussion

In this section we present and discuss the results of our experimental analysis evaluating and comparing Survival-LCS.

### 3.1 Predictive Performance

First we focus on the evaluations and comparisons of predictive performance based on model IBS scores.

#### 3.1.1 Comparing Survival-LCS to CPH.

Survival-LCS results summarized in Tables 4-11 give mean IBS scores for simulations involving the four genetic models (additive, epistatic, heterogeneous, and univariate, respectively). The IBS scores for CPH Models, where applicable, are shown in conjunction with all Survival-LCS models run on 100 features in Tables 4, 6, 8, and 10. The Survival-LCS runs for 1,000 and 10,000 features are shown in Tables 5, 7, 9, and 11. For all tables we compare the previous Random Spline distribution runs (which used only 50,000 iterations and a maximum rule population size of 1,000) [Woodward et al., 2024] to new runs with Random Spline, Gamma, Gaussian, and Weibull baseline survival distributions (using 200,000 iterations and a max rule population size of 2,000).

The left-hand columns in these tables indicate the respective simulated dataset characteristics, and the right-hand columns give IBS values for the different baseline distributions, i.e., Weibull, Gamma, Gaussian, and **Random Monotonic Spline (RS)**. For datasets with 100 features, permutation testing (with 100 permutations) was conducted to validate the performance of Survival-LCS empirically. Shaded values represent models that are performing relatively well, i.e., making fairly accurate predictions compared to a “non-informative” model that predicts the overall event rate in a 50% incidence of the event scenario (IBS < 0.25). IBS scores of less than 1, a known artifact of integration by a trapezoidal rule in certain distributions [NumPy-Developers, 2014], are represented as “NA” in the paper for clarity. If an “NA” is present, no corresponding bold highlighting is done. Values with an asterisk indicate significant results (p < 0.05) based on permutation testing, while bold values indicate whether Survival-LCS or CPH performed better (based on having a lower mean IBS).

Examining the results of datasets with only 100 features in Tables 4, 6, 8, and 10, we observe that Survival-LCS achieved comparable IBS scores to CPH Models except for some cases of high censoring. The confidence intervals of the IBS values in Survival-LCS and CPH models overlapped in most cases (see Supplementary Tables S6-S25), limiting definitive conclusions about superior performance but largely supporting comparative performance. In terms of Brier score, the differences were also statistically significant (p < 0.05) compared to the null distributions generated via permutation testing. Of note, we refrain from making definitive claims about the Weibull distribution results, as several IBS scores fell in the negative range—an artifact that can occur when using trapezoidal integration on certain survival curves [NumPy-Developers, 2014].

In high-dimensional settings (e.g., datasets with 1,000 or 10,000 features), CPH models fail to run due to computational and scalability constraints. In contrast, Survival-LCS consistently handled these high-dimensional datasets with stable IBS scores (<0.25), demonstrating its robustness except in cases of high censoring proportions (i.e., 0.8). While there are regularized variants of the CPH model addressing dimensionality challenges [Bradic et al., 2011; Guo and Yi, 2022], they still rely on strict assumptions, unlike Survival-LCS, which not only avoids assumptions but was also implemented to accommodate missing data values without requiring imputation. This is an advantage natural to many rule-based machine learning algorithms [Lipschutz-Villa et al., 2025; Urbanowicz and Browne, 2017; Urbanowicz and Moore, 2015].

More closely comparing the original random spline results (i.e., RS 50k, 1k) [Woodward et al., 2024] with the new baseline distribution datasets using more generous Survival-LCS hyperparameters settings, we observed that Survival-LCS performs fairly consistently across the different survival scenarios, including higher feature counts and more complex genetic models (i.e., heterogeneous). While some distributions performed better than others, across all models, Survival-LCS exhibited reliable predictive performance, underscoring its potential for application in diverse real-world survival analyses, particularly in higher-dimensional, noisy, or data with missing values where the applicability of traditional methods is limited. These results also highlight challenges faced by Survival-LCS on datasets with skewed distributions of censoring times, similar to the results previously observed with skewed survival distributions and censoring times in our initial analysis [Woodward et al., 2024].

#### 3.1.2 Comparing Survival-LCS Performance across Different Survival Dataset Configurations.

Further statistical analyses using the Wilcoxon Rank Sum Test were applied to examine differences in mean IBS for Survival-LCS (given in Supplementary Tables S6-S25) across different genetic models, MAFs, total number of features, and censoring proportions to examine how each impacted performance. These comparisons were completed for each of the 4 simulated survival distributions using the extended hyperparameters settings in this study and contrasted with the previous findings using only random spline and the limited hyperparameter settings in Woodward et al. [2024]. These individual comparisons are given in Supplementary Tables S26-S45.

With respect to the different genetic models, our previous findings [Woodward et al., 2024] using the random spline survival distribution and limited hyperparameter settings showed significant differences, with the additive and epistatic datasets yielding better performance than the univariate datasets (p = 0.0120, p = 0.0483, respectively) and the best performance overall. The additive datasets also outperformed the heterogeneous datasets (p = 0.0483). When increasing the hyperparameter settings to 200,000 iterations and a maximum rule population size of 2,000 but keeping the random spline distribution, only one significant performance difference was observed, where performance on additive datasets was better than the univariate datasets (p = 0.0303). Similarly, no significant difference between performance on the genetic models was observed for the Gamma or Gaussian distributions, and only the performance on the epistatic datasets was found to outperform heterogeneous datasets for the Weibull distribution. This suggests that Survival-LCS was able to perform more consistently over genetic models given more computing resources. Of note, heterogeneous datasets are known to be particularly challenging to tackle, as they include predictive variables that are only predictive in a given subset of training instances. Although performance on heterogeneous datasets was significantly worse in the original study, our findings suggest that this can be at least partially mitigated by using a higher number of learning iterations during training to tackle the more challenging data problem, further highlighting the importance of this LCS hyperparameter.

With respect to the total number of features, the previous analysis yielded no significant differences between performance on datasets 100, 1,000, and 10,000 features [Woodward et al., 2024]. This held true for increased hyperparameter settings for all distributions with the exception of Weibull, where we observed better performance (i.e., lower IBS) in datasets with fewer features (100 vs. 1,000 features, p = 0.001; 100 vs. 10,000 features, p = 0.009).

With respect to censoring, across all survival distributions and hyperparameter settings, having lower censoring yielded significantly better performance, as would be expected since censoring adds uncertainty.

And lastly, with respect to MAF, while across all survival distributions and hyperparameter settings, no significant performance differences were observed, we did note a slightly greater impact on performance when using the extended hyperparameter settings in contrast with the previous analyses [Woodward et al., 2024].

#### 3.1.3 Examination of Survival-LCS-Predicted Survival Probability Distributions.

**Fig. 4.**
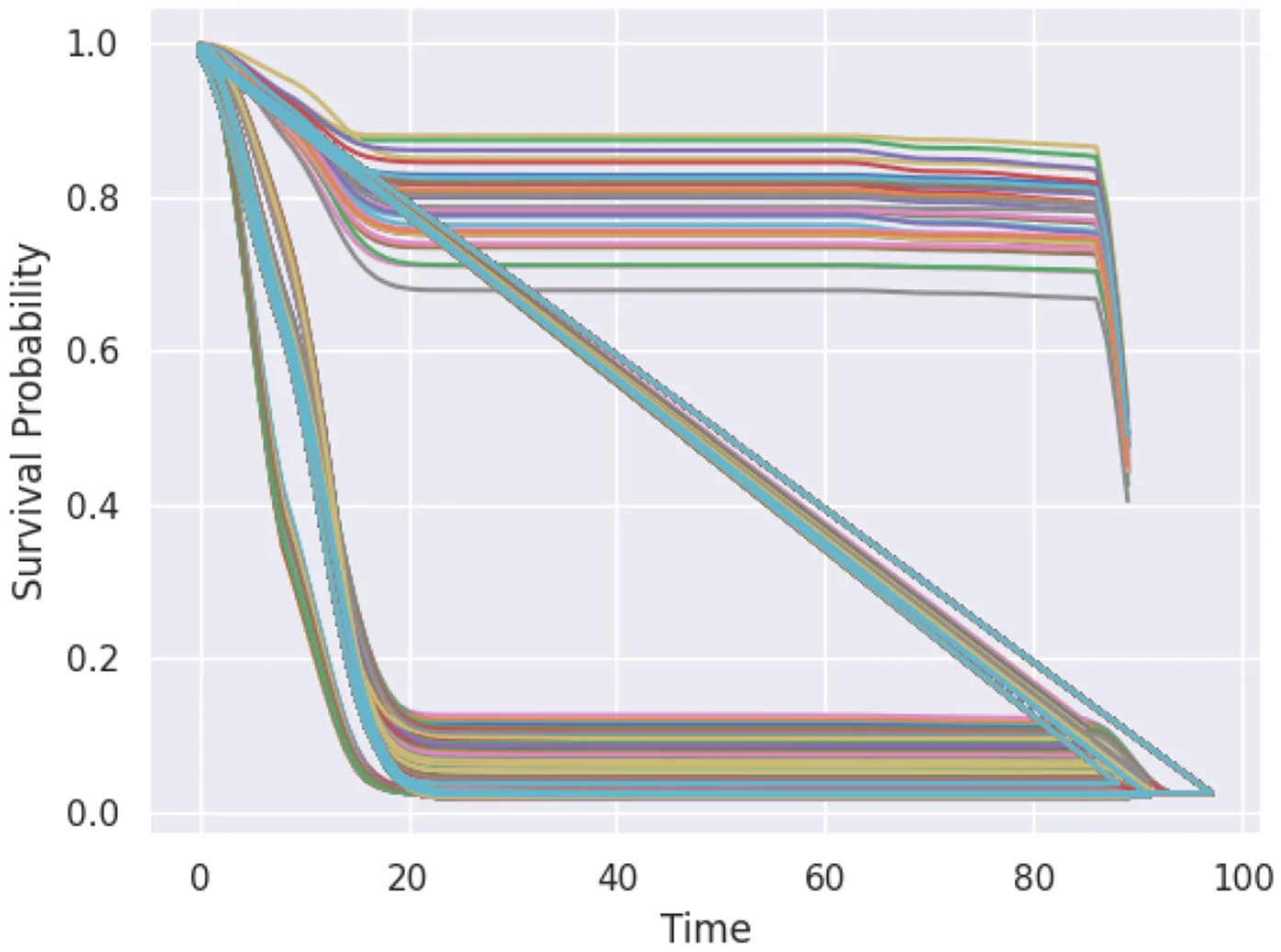
Predicted survival probability distributions for 1,000 instances in a dataset simulated with an underlying epistatic genetic model with MAF = 0.2, censoring = 0.4, number of features = 1,000, and a gamma baseline survival time distribution.

Figure 4 depicts an example of predicted survival probability distributions generated by Survival-LCS for a simulated survival dataset with an underlying epistatic genetic model. Each line in the figure represents the predicted survival probability distribution for an individual instance. Notably, distinct groups of lines emerge, reflecting similarly shaped survival probability distributions. These groupings highlight distinct survival behaviors among instances and include: (1) A gradual decline followed by a sustained high survival probability: Instances where survival probability decreases less abruptly at first, levels out for the majority of the time window, and then sharply drops to non-zero probability later in the time window. (2) Straight Diagonal Lines: Representing instances with low rule coverage, where the **probability density function (PDF)** estimation was inadequate. These lines indicate a constant rate of survival probability that decreases over time, arising from insufficient data support in the rule set. (3) Sharp Drop to Zero: A subset of instances where survival probability rapidly declines to nearly zero over a short time frame, then sustains it for nearly the complete time frame before dropping to zero.

Except for the diagonal lines, these groupings correspond to some of the different simulated epistatic genotype combinations. The clustering of these survival distributions reflects the underlying allele frequency and penetrance values used to generate the epistatic association. Table 3 provides the penetrance table used to generate this dataset, which details the underlying genotype-to-survival-time mapping. The correspondence between the number of lines in each grouping and allele frequency, as well as the decline in survival curves as a reflection of penetrance values in Table 3, highlights Survival-LCS’s ability to capture key parts of this underlying mapping in the dataset. These survival probability distributions offer a direct visualization of the complex relationships between genotypes and survival outcomes.

### 3.2 Run Time Assessment

**Fig. 5.**
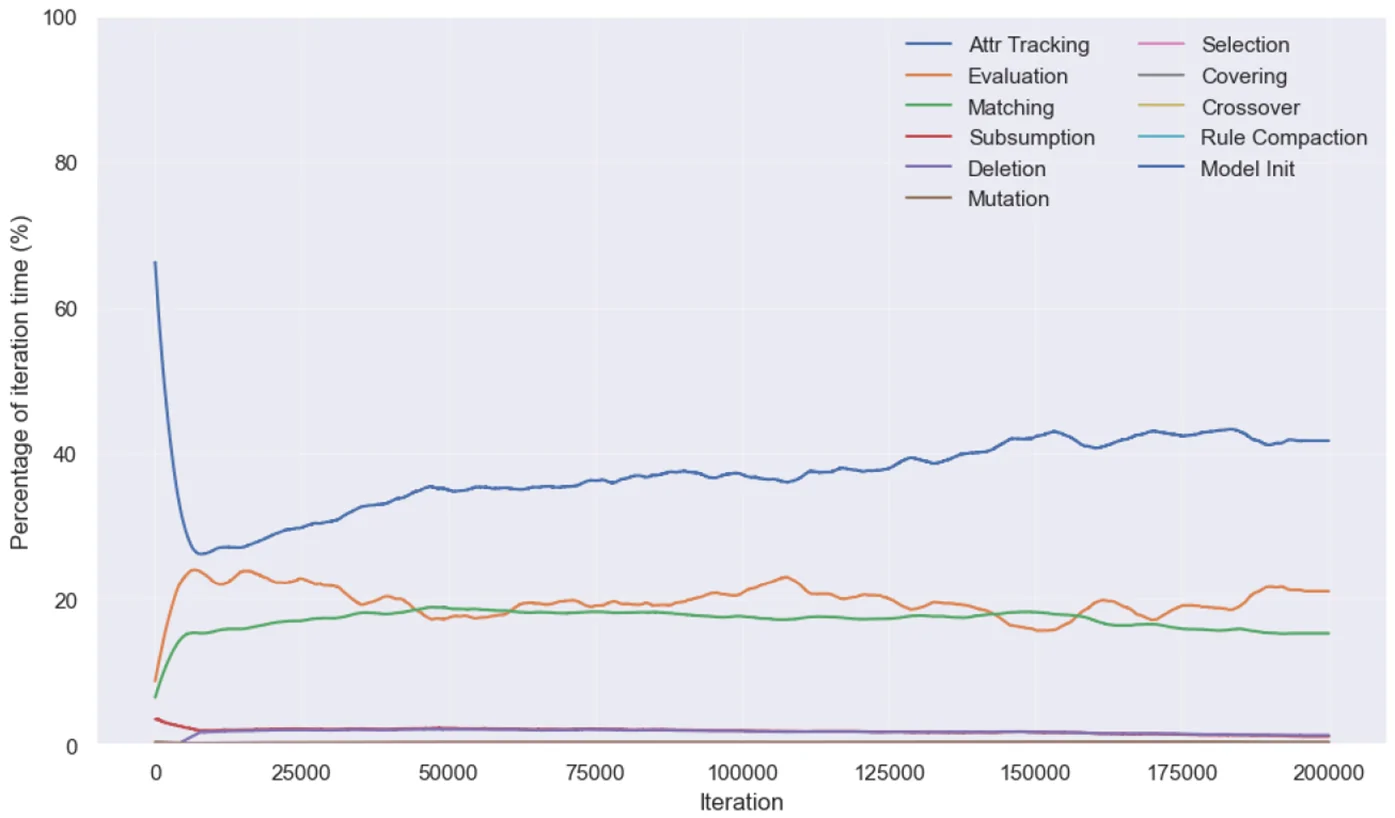
Plot of the runtime percentage (across learning iterations) for each operation type during a single CV run on a random spline heterogeneous dataset (10,000 features; MAF = 0.2; censoring = 0.1).

With respect to Survival-LCS runtime, the total number of features in the dataset had the largest impact, as would be expected. Time complexity is also impacted by the number of instances, the number of training iterations, and the maximum size of the rule population. Figure 5 plots the percentage of per-iteration runtime spent in each Survival-LCS component. After an initial warm-up spike, the composition is largely stable. Attribute tracking accounts for the largest share, followed by rule evaluation, and matching. All other operations, including selection, covering, crossover, mutation, deletion, subsumption, and rule compaction, each contribute only a few percent per iteration and remain nearly flat. This makes attribute tracking, rule evaluation and matching potential targets for algorithm efficiency improvements in the future. Across different dataset configurations we observe similar profiles, indicating a relatively steady sharing of runtimes. Total runtimes varied, with random spline distribution experiments generally taking the most runtime.

### 3.3 Feature Importance, Model Visualizations, and Model Interpretability

Previous work in LCS has introduced an attribute-tracking strategy to estimate feature importance on a per-instance basis. This method relies on quantifying the number of times a particular feature is referenced in the rules comprising a given [C] [Urbanowicz et al., 2012a]. By aggregating these counts, attribute tracking provides a measure of how frequently a feature is utilized across the rule set, offering insights into its relative importance. In the context of Survival-LCS, this strategy has proven effective in identifying simulated predictive features across various underlying genetic models. Specifically, features with the highest attribute tracking scores or those specified in rules with superior predictive accuracy were often correlated with the true predictive features. However, this approach is not without limitations. One notable drawback is its inability to account for the direction of effect, such as whether a specific feature value is associated with a positive or negative impact on the outcome.

**Fig. 6.**
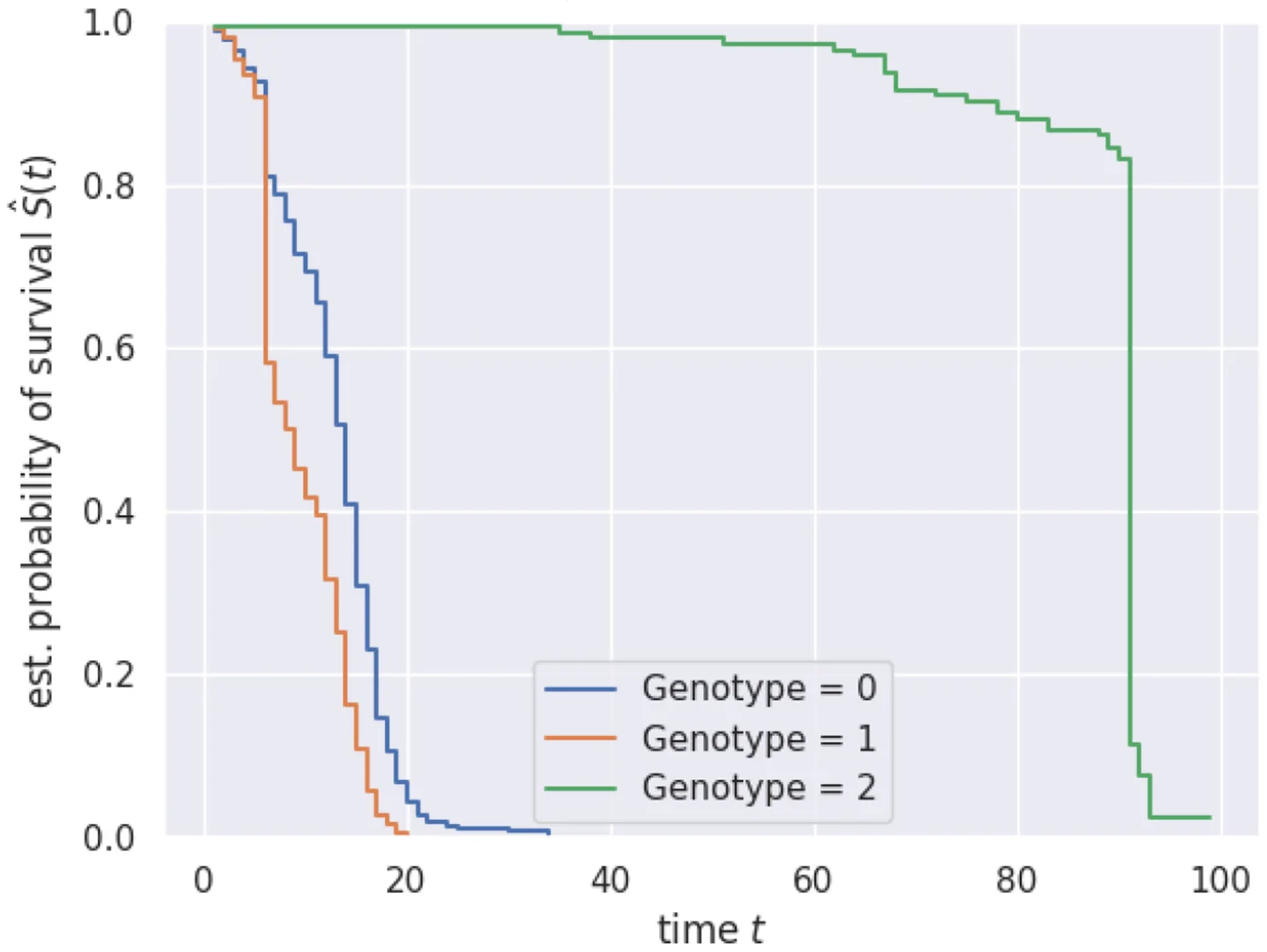
Kaplan-Meier plot of the simulated genetic feature (M0P2) from a univariate dataset generated with a gamma distribution, MAF = 0.2, censoring = 0.1 and number of features = 10,000. Note how the three possible genotypes states correspond to distinct survival probability curves.

To address this limitation, Survival-LCS provides an additional feature visualization tool: the ability to generate **Kaplan–Meier (KM)** survival plots based on user-specified top-ranking features. This allows users to not only identify important features but also directly observe their relationship with survival outcomes. For example, in Figure 6, the top feature identified by attribute tracking, “M0P2,” was confirmed as the true predictive feature in an analyzed univariate dataset. The KM plot reveals the direction of the effect associated with different genotypes of “M0P2”: a genotype of 2 is protective, while genotypes 0 and 1 are associated with a reduced probability of survival. This dual approach, i.e., leveraging attribute tracking scores to identify important features and interrogate the impact of a given feature’s states on survival probabilities over time, offers a more nuanced understanding of feature importance and its implications in survival analysis.

**Fig. 7.**
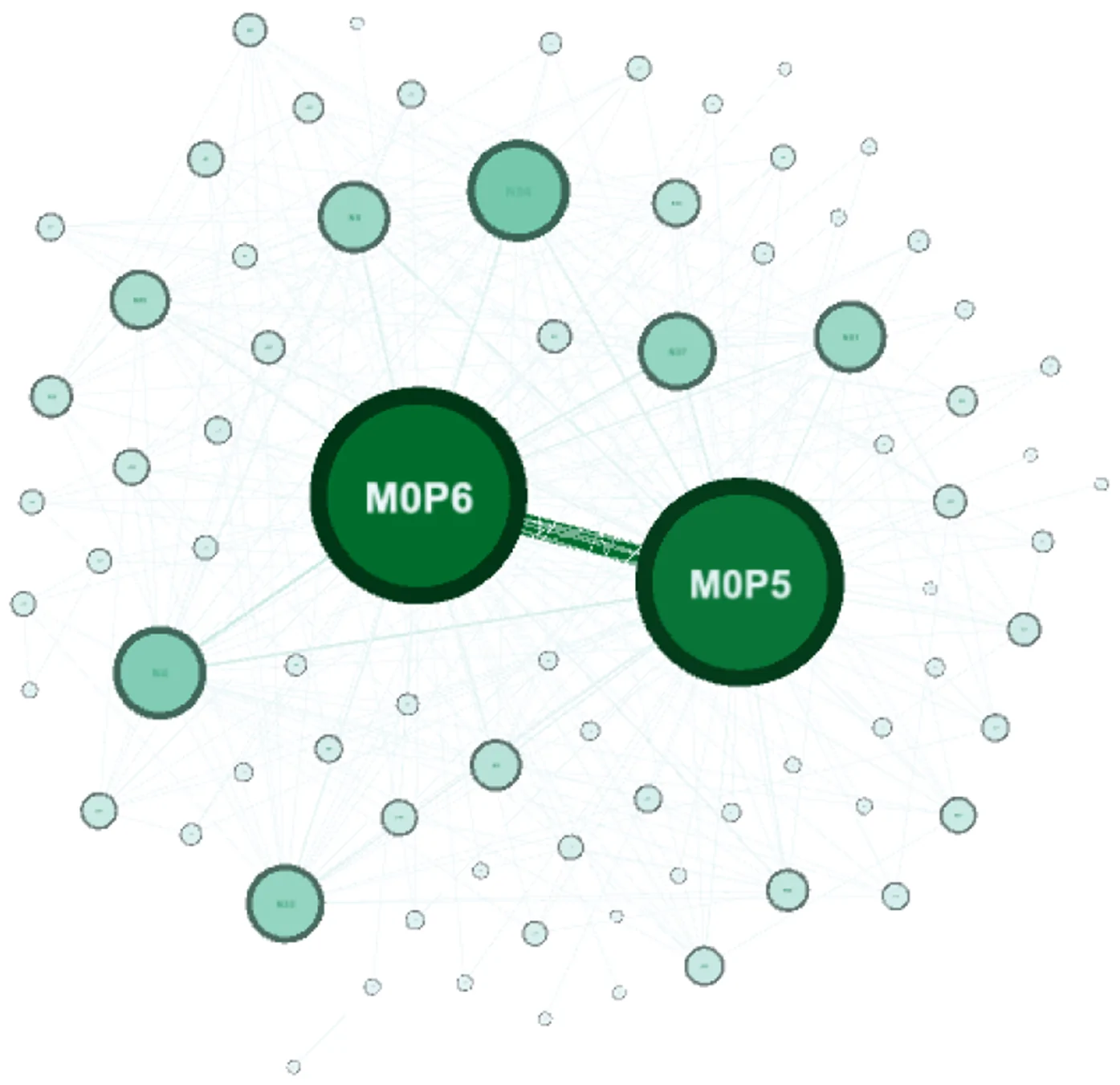
Feature co-occurrence network of a trained Survival-LCS model using a simulated dataset with an epistatic genetic model, a gamma survival distribution, 100 features, MAF = 0.2, and censoring = 0.1. Node diameter is proportional to the frequency of a feature’s occurrence in the rule population. Edges correspond to the frequency of co-specification of pairs of features in each rule. Labeled nodes (M0P5 and M0P6) are the simulated, truly predictive, epistatic single-nucleotide polymorphisms (SNPs).

In a separate example of Survival-LCS model interpretability, we show that the interacting features from a simulated epistatic dataset are correctly highlighted within a feature co-occurrence network [Urbanowicz et al., 2012b] generated by LCS-Dive [Zhang et al., 2021] based on a trained Survival-LCS rule population [P]. As seen in Figure 7, predictive features M0P5 and M0P6 (which were simulated to have a pure epistatic interaction between them that was predictive of survival time) were correctly identified as interacting features. These features were frequently co-specified in rules within the trained [P], indicating their combined relevance to the modeled outcome. The network visualization employs two key attributes to convey the significance of features and their interactions. Node size corresponds to the total number of times a feature was specified in the rules across [P], reflecting its individual importance within the rule set. Edge size, on the other hand, represents the frequency with which pairs of features were simultaneously specified in the rules, providing insights into potential epistatic interactions. Together, these visual elements enable users to quickly assess the relative importance of features, the strength of feature interactions, and the heterogeneity of associations captured by the rule population in [P]. These network visualizations can facilitate rapid visual interpretation of feature importance, epistatic interactions, and heterogeneous associations of a large population of rules in [P] [Urbanowicz et al., 2012b].

**Fig. 8.**
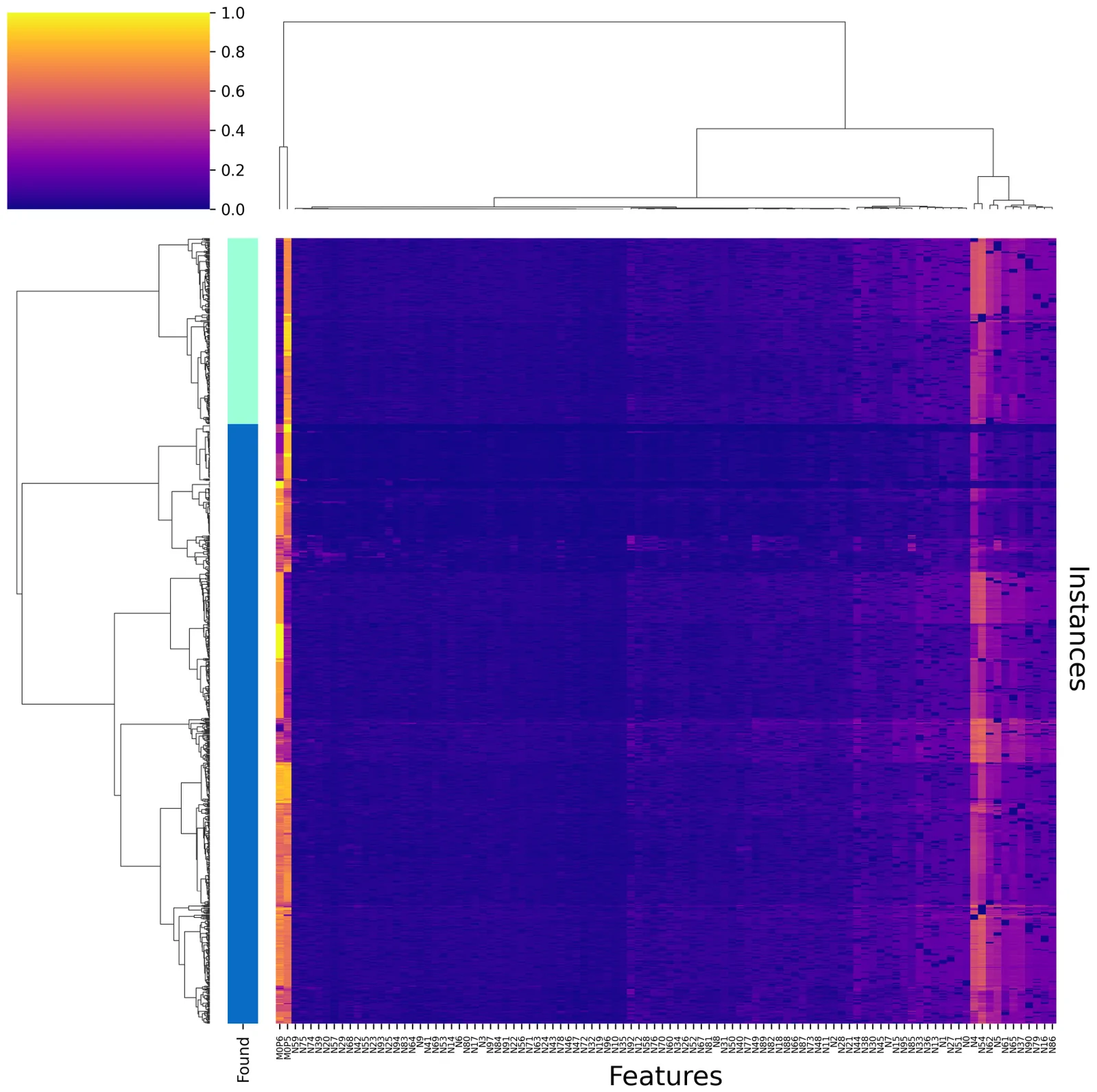
Clustered heatmap of normalized attribute tracking scores taken from a Survival-LCS model trained on simulated data with an epistatic genetic model, a gamma distribution, and 100 total features. These attribute tracking scores were merged across all CV partitions (using LCS-Dive). Brighter heatmap colors indicate higher attribute tracking scores, and darker values indicate a lower score. Both features and instances are clustered using hierarchical clustering. Two significant clusters of instances are identified by the left vertical bar labeled “Found.”

A final example visualization highlights the ability of Survival-LCS to characterize underlying multivariate associations. Using an epistatic dataset with 100 features, hierarchical clustering was performed on the attribute tracking scores across training instances. This analysis successfully identified significant subgroups, as visualized in Figure 8. The clustering revealed two distinct instance groups, distinguished by the “Found” column, which were identified through LCS-Dive’s automated clustering approach. The larger cluster was primarily driven by the two interacting features, “M0P5” and “M0P6.” Of note, this heatmap visualization and identification of the underlying epistatic interaction was much clearer in the present study (using a larger number of training iterations) than that reported in the preliminary work [Woodward et al., 2024] again highlighting the value of optimizing Survival-LCS run parameters (i.e., training iterations and max rule population size).

Lastly, Table 12 shows the top 10 rules evolved by Survival-LCS (ranked by rule numerosity) for the same simulated dataset with an epistatic genetic model, a gamma distribution, and 100 total features. This provides an example of what a Survival-LCS model looks like at the individual rule level and highlights both the fundamental interpretability of rule-based machine learning models and how the individual rules in this scenario are able to correctly capture an epistatic relationship between two predictive features (i.e., M0P5 and M0P6). Note how almost all of the top 10 rules correctly specify both “M0P6” and “M0P5” as features and successfully avoid specifying other non-predictive features from the data. These rules also map the value states of these specified features to predicted event intervals of survival time. This top ruleset much more clearly captures the underlying relationships in this model than observed in previous work with limited hyperparameter settings [Woodward et al., 2024]. This demonstrates that Survival-LCS is able to focus on the relevant attributes without introducing spurious conditions (such as overfitting), demonstrating both effective feature selection and model parsimony under multi-objective pressures. The “Iteration Initialized” column of Table 12 also reveals that most of the highest accuracy rules were discovered after 95k iterations. This supports the expectation that a larger number of training iterations helped improve the performance of survival-LCS in comparison to the original analysis. Determining the ideal relationship between model complexity and algorithm iterations required could be an additional avenue for future work. Alternatively, utilizing alternative strategies to rank rules in [P] might also help to address the challenge of characterizing underlying patterns of association in survival data in future work.

### 3.4 Limitations

Despite its demonstrated strengths, Survival-LCS has certain limitations that merit further investigation. One notable limitation is its performance under conditions of extreme censoring, where the reduced availability of event data impacts its ability to accurately model survival outcomes, particularly in datasets with heterogeneous structures. While Survival-LCS effectively leverages censored data up to moderate censoring proportions, high censoring introduces challenges in rule generalization and predictive accuracy. This may be a fundamental limitation on such datasets; however, it is possible that algorithmic improvements could handle the uncertainty of high censoring levels more effectively.

**Fig. 9.**
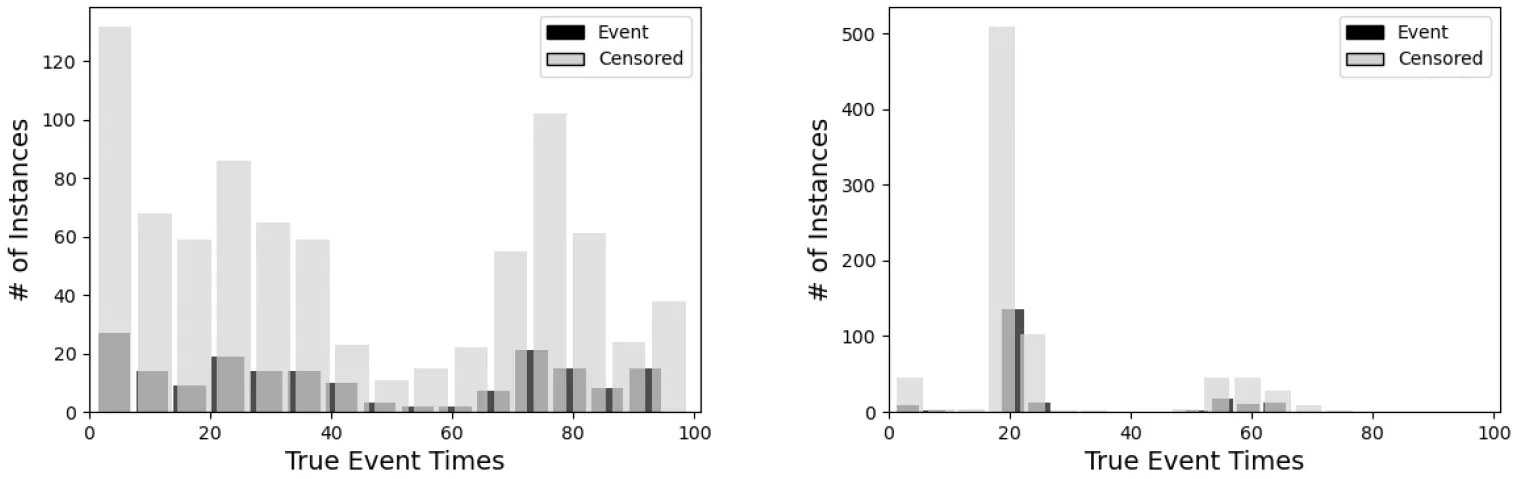
Well-distributed vs. skewed censored survival times impact Survival-LCS performance. On the left we show a well-performing Survival-LCS run on data with a gamma baseline distribution (IBS = 0.17). On the right we show a poor-performing Survival-LCS run on data with a Gaussian distribution (IBS = 0.51). In both Survival-LCS trained on a simulated dataset with an additive model, 1,000 features, MAF = 0.4, and censoring = 0.8.

Also, with the added examination of different baseline survival distributions in this study, we observed some poor IBSs (>0.25) on some datasets, mostly for higher values of censoring. Figure 9 shows the difference in the distribution of survival times and events of well-performing and poor-performing models. While, by design, the event times are not randomly distributed, the impact of the final censoring distribution should be considered (as it is still randomly picked in our analysis). Skewed censoring time seems to adversely affect the performance of Survival-LCS. This limitation highlights potential areas for refinement, such as incorporating mechanisms to better leverage limited event data or improving robustness under high uncertainty.

Further, the computational demands of Survival-LCS represent another potential limitation. While increasing the rule population size and training iterations improves model performance, it also significantly raises the computational burden. This tradeoff could pose challenges when applying Survival-LCS to extremely large datasets or in environments requiring rapid decision-making. Addressing these demands through optimization techniques or hardware-based solutions could expand the algorithm’s practical applicability in resource-constrained settings.

Additionally, while Survival-LCS exhibits strong performance compared to traditional methods like CPH and has inherent advantages with respect to avenues for model interpretation, this study has not yet sought to directly compare predictive performance with other survival-data-focused machine learning approaches such as RSFs or deep learning. While these algorithms are not expected to be capable of achieving similar interpretability, they could potentially have advantages in scalability, efficiency, and predictive performance (under certain scenarios). Future studies will systematically compare our rule-based approach with such methods across a variety of scenarios to fully characterize relative strengths and limitations.

We also note that while Survival-LCS is based on the ExSTraCS algorithm, a new, much more effective rule-based machine learning approach was recently developed called **HEROS (Heuristic Evolutionary Rule Optimization System)** [Lipschutz-Villa et al., 2025]. HEROS takes elements from ExSTraCS and a number of other proposed LCS algorithms, laying out a two-phase multi-objective approach to rule-based modeling to yield highly accurate and compact rule sets for maximal interpretability. In future work we plan to adapt the algorithmic concepts introduced in Survival-LCS in order to expand the HEROS framework to survival analysis applications.

Finally, while this study focuses on simulated datasets to control for ground truth and systematically evaluate performance across a wide range of conditions (e.g., survival distribution, censoring, genetic models) as a proof of concept, future work will apply our survival analysis capable, rule-based machine learning algorithms to real-world biomedical datasets to further assess practical utility and generalizability.

## 4 Conclusions

Increasing examples in the survival literature have demonstrated that machine-learning-based survival methods can outperform CPH and other traditional models [Li et al., 2023; Moncada-Torres et al., 2021]. This study extends original work conducted by Woodward et al. [2024], introducing and demonstrating the proof-of-principle efficacy and interpretability of Survival-LCS as a tool for machine learning-driven survival analysis, particularly in complex and/or higher-dimensional scenarios. Unlike traditional methods such as the CPH model, Survival-LCS circumvents assumptions of PH and linearity, allowing it to model nonlinear, epistatic, and heterogeneous patterns with greater flexibility. Furthermore, this rule-based survival algorithm is compatible with categorical and quantitative feature types, handles missing values in training or testing data, shows promise in scaling up to higher-dimensional data, and has the fundamental interpretability of a model comprised of IF:THEN expressions, underscoring its potential for real-world application.

The expanded evaluation presented here examined additional baseline survival distributions and extended algorithm hyperparameter settings, which further supports the scalability of Survival-LCS to datasets with at least 10,000 features and a range of censoring proportions. Survival-LCS maintained consistent performance across a variety of simulated dataset configurations, offering reliable predictions and demonstrating resilience in high-dimensional contexts where traditional survival analysis methods falter. Nonetheless, the computational cost of training remains a practical constraint, as well as reduced accuracy in cases of extreme censoring. Enhancing the algorithm to mitigate these challenges could broaden its applicability and ensure its utility across a wider range of survival analysis tasks.

Overall, Survival-LCS stands out as a promising approach that blends interpretability, flexibility, and robustness. Its potential to uncover complex survival patterns in biomedical and other domains underscores its value as a powerful tool for advancing research and informing practical decision-making. Future efforts could also build upon this method by (1) improving code efficiency and ease of use for real-world applications, (2) examining adaptations to handle left and interval censoring, and (3) incorporating a weighting approach similar to inverse probability of censoring weights to account for bias from dependent censoring. This work has demonstrated that rule-based machine learning algorithms can be successfully adapted to survival and other time-to-event analyses in a data-driven manner, in particular when the assumptions required by other approaches cannot be satisfied.

## Supplementary Material

Supplementary Material

## Figures and Tables

**Table 1.** Error Updates for Rules in [C] and [I] Given Censored or Uncensored Instances

	Event Occurred	Censored
[C]	$\text{Err}=\frac{|EI_{C}-evenTime|}{T_{\max}-T_{\text{min}}}$	Err=0
[I]	Err=1	Err=1×propIncorrect

**Table 2.** Simulated Data Parameter Values Examined in This Study

Parameter	Values
Baseline Distribution	Random Spline, Gaussian, Gamma, Weibull
Genetic Model	Additive, Heterogeneous, Epistatic, Univariate
Number of Features	100, 1,000, 10,000
Censoring Proportion	0.1, 0.4, 0.8
Model Allele Frequency	0.2, 0.4

A full factorial study design was used, yielding a total of 288 unique simulated datasets (4 × 4 × 3 × 3 × 2).

**Table 3.** Epistatic Penetrance Table Generated by GAMETES

		Feature_1		
Feature_2		0	1	2
	0	0.621	0.337	0.726
	1	0.329	**0.995**	0.140
	2	0.794	**0.005**	0.0619

The genotype combination where feature 1 is 1 and feature 2 is 1 yields the highest simulated survival times, while the combination where feature 1 is 1 and feature 2 is 2 yields the smallest survival times.

**Table 4.** Additive Datasets (100 Features)—IBSs across Different Survival Distributions for sLCS and CPH

MAF	Censoring	Model	RS (50k, 1k)	RS	Gamma	Gaussian	Weibull
0.2	0.1	sLCS	0.0560	**0.0780** *	**0.1035** *	0.1020*	0.0434
		CPH	**0.0534**	0.0837	0.1036	**0.0999**	NA
	0.4	sLCS	0.1752	0.1647*	0.1192	0.1114	0.1681
		CPH	**0.1082**	**0.1266**	**0.1053**	**0.0813**	NA
	0.8	sLCS	0.1232*	0.1902*	0.4375	0.3629*	0.2596
		CPH	**0.0868**	**0.1522**	**0.1992**	**0.1919**	**0.1731**
0.4	0.1	sLCS	0.1523	0.0888*	**0.1176** *	0.1622	0.1418
		CPH	**0.1238**	**0.0789**	0.1240	**0.1004**	NA
	0.4	sLCS	0.1206	0.1721	0.1454	0.2252	0.1581
		CPH	**0.1081**	**0.1694**	**0.1124**	**0.1517**	NA
	0.8	sLCS	0.2198	0.3292	0.5023	0.3395	0.0885*
		CPH	**0.1528**	**0.2203**	**0.2331**	**0.1790**	NA

Shaded cells indicate IBS < 0.25. Bold values represent the best-performing model (lower mean IBS), and an asterisk (*) indicates a statistically significant difference (p < 0.05) based on a permutation test.

**Table 5.** Additive Datasets (1,000 and 10,000 Features)—IBSs for sLCS across Different Survival Distributions

Features	MAF	Censoring	RS (50k, 1k)	RS	Gamma	Gaussian	Weibull
1,000	0.2	0.1	0.0779	0.1148	0.0547	0.1142	0.1567
		0.4	0.2069	0.0971	0.1107	0.1713	0.1604
		0.8	0.3978	0.3482	0.3951	0.2835	0.2789
	0.4	0.1	0.1295	0.1026	0.0424	0.1307	0.1841
		0.4	0.1435	0.1250	0.2821	0.1110	0.1303
		0.8	0.4339	0.3689	0.1723	0.5137	NA
10,000	0.2	0.1	0.0409	0.0646	0.1230	0.0775	0.0898
		0.4	0.1730	0.1672	0.3659	0.1769	0.1908
		0.8	0.4000	0.3699	0.3898	0.5372	0.4134
	0.4	0.1	0.1058	0.0923	0.0993	0.1163	0.1405
		0.4	0.0963	0.2120	0.0845	0.2003	0.1731
		0.8	0.0844	0.4601	0.4787	0.4140	0.5464

Shaded cells indicate IBS < 0.25.

**Table 6.** Epistatic Datasets (100 Features)—IBSs across Different Survival Distributions for sLCS and CPH

MAF	Censoring	Model	RS (50k, 1k)	RS	Gamma	Gaussian	Weibull
0.2	0.1	sLCS	**0.0733**	**0.1264** *	**0.1138** *	**0.1208** *	**0.0753**
		CPH	0.0763	0.1631	0.1352	0.1249	0.1504
	0.4	sLCS	0.2011	**0.1158** *	0.1254	**0.1461** *	**0.1582**
		CPH	**0.1780**	0.1380	**0.1049**	0.1733	0.2157
	0.8	sLCS	0.3084	0.1331	0.2591	1.1466	**0.0994**
		CPH	**0.1960**	**0.1053**	**0.1404**	**0.3426**	3.5129
0.4	0.1	sLCS	**0.1553** *	**0.1028** *	**0.1285** *	**0.1225** *	**0.1964**
		CPH	0.1761	0.1594	0.1488	0.1545	0.2433
	0.4	sLCS	0.2831	0.1632	0.1791	**0.1239**	0.1039
		CPH	**0.2354**	**0.1629**	**0.1701**	0.1256	NA
	0.8	sLCS	0.1765	0.2674	0.1729	0.4070	0.3358
		CPH	**0.1527**	**0.1649**	**0.1136**	**0.2181**	NA

Shaded cells indicate IBS < 0.25. Bold values represent the best-performing model (lower mean IBS), and an asterisk (*) indicates a statistically significant difference (p < 0.05) based on a permutation test.

**Table 7.** Epistatic Datasets (1,000 and 10,000 Features)—IBSs for sLCS Across Different Survival Distributions

Features	MAF	Censoring	RS (50k, 1k)	RS	Gamma	Gaussian	Weibull
1,000	0.2	0.1	0.2336	0.1437	0.0836	0.1171	0.0832
		0.4	0.1420	0.1819	0.1273	0.2109	0.2023
		0.8	0.1847	0.3559	0.3578	0.4373	0.3124
	0.4	0.1	0.0871	0.1308	0.1883	0.0965	0.1615
		0.4	0.1496	0.1600	0.2820	0.1136	0.1378
		0.8	0.4093	0.1972	0.4617	0.1745	0.4226
10,000	0.2	0.1	0.0727	0.1596	0.0664	0.0800	0.1443
		0.4	0.1340	0.1765	0.2152	0.1793	0.1409
		0.8	0.1490	0.4128	0.1107	0.4548	0.2968
	0.4	0.1	0.1055	0.1879	0.1291	0.1483	0.1072
		0.4	0.2470	0.1175	0.2439	0.1265	0.1142
		0.8	0.5548	0.0722	0.3571	0.4963	0.3429

Shaded cells indicate IBS < 0.25.

**Table 8.** Heterogeneous Datasets (100 Features)—IBSs across Different Survival Distributions for sLCS and CPH

MAF	Censoring	Model	RS (50k, 1k)	RS	Gamma	Gaussian	Weibull
0.2	0.1	sLCS	**0.1415**	0.1799	0.2484	**0.1914** *	0.0821
		CPH	0.1455	**0.0889**	**0.2006**	0.2131	NA
	0.4	sLCS	0.1854*	**0.1228**	**0.1185** *	0.1966	**0.0966**
		CPH	**0.1805**	0.1654	0.1188	**0.1717**	0.1420
	0.8	sLCS	0.3686	0.2275	1.2000	0.4542	**0.2047**
		CPH	**0.1727**	**0.1267**	**0.2956**	**0.1701**	0.2959
0.4	0.1	sLCS	0.0929	0.1617	0.1449*	**0.0727** *	0.0976
		CPH	**0.0891**	**0.1411**	**0.1421**	0.0746	NA
	0.4	sLCS	**0.1366**	**0.2007**	0.2266	0.2106	0.1099
		CPH	0.1388	0.2444	**0.1929**	**0.1992**	NA
	0.8	sLCS	0.8103	0.3699	0.3459	0.2457	0.5167
		CPH	**0.2572**	**0.1782**	**0.2120**	**0.1719**	NA

Shaded cells indicate IBS < 0.25. Bold values represent the best-performing model (lower mean IBS), and an asterisk (*) indicates a statistically significant difference (p < 0.05) based on a permutation test.

**Table 9.** Heterogeneous Datasets (1,000 and 10,000 Features)—IBSs for sLCS across Different Survival Distributions

Features	MAF	Censoring	RS (50k, 1k)	RS	Gamma	Gaussian	Weibull
1,000	0.2	0.1	0.2829	0.1131	0.1092	0.1348	0.1943
		0.4	0.1379	0.1484	0.1590	0.3096	0.1562
		0.8	0.2707	0.2525	0.2335	0.4689	0.3896
	0.4	0.1	0.1497	0.1011	0.1693	0.1487	0.1635
		0.4	0.1382	0.1556	0.2196	0.1700	0.2212
		0.8	0.3447	0.2075	0.1759	0.1682	0.4297
10,000	0.2	0.1	0.1143	0.1714	0.0609	0.2072	0.2803
		0.4	0.2956	0.1347	0.2101	0.2500	0.2437
		0.8	0.4537	0.1030	0.4085	0.2575	0.3534
	0.4	0.1	0.1226	0.2418	0.0678	0.0799	0.0885
		0.4	0.2320	0.2022	0.2547	0.1762	0.2156
		0.8	0.2023	0.3066	0.2042	0.2042	0.2893

Shaded cells indicate IBS < 0.25.

**Table 10.** Univariate Datasets (100 Features)—IBSs across Different Survival Distributions for sLCS and CPH

MAF	Censoring	Model	RS (50k, 1k)	RS	Gamma	Gaussian	Weibull
0.2	0.1	sLCS	**0.1587** *	0.1299*	0.2645	0.0904	0.2251
		CPH	0.1659	**0.0897**	**0.1476**	**0.0548**	NA
	0.4	sLCS	0.1797	0.1604*	0.3426	0.1939	0.1655
		CPH	**0.1100**	**0.1104**	**0.2307**	**0.0979**	NA
	0.8	sLCS	0.3882	0.3428	0.4421	0.3383	0.2762*
		CPH	**0.2542**	**0.2520**	**0.1710**	**0.1557**	**0.2000**
0.4	0.1	sLCS	0.1573*	0.1376*	0.1344*	0.0989*	0.0975
		CPH	**0.0687**	**0.0720**	**0.1074**	**0.0551**	**0.0971**
	0.4	sLCS	0.1781*	0.1668*	0.1672*	0.1654*	0.1372
		CPH	**0.0999**	**0.0746**	**0.0989**	**0.0869**	NA
	0.8	sLCS	0.2407	0.3185*	0.2767	0.2118*	**0.1540**
		CPH	**0.1460**	**0.0972**	**0.1544**	**0.1092**	0.1723

Shaded cells indicate IBS < 0.25. Bold values represent the best-performing model (lower mean IBS), and an asterisk (*) indicates a statistically significant difference (p < 0.05) based on a permutation test.

**Table 11.** Univariate Datasets (1,000 and 10,000 Features)—IBSs for sLCS across Different Survival Distributions

Features	MAF	Censoring	RS (50k, 1k)	RS	Gamma	Gaussian	Weibull
1,000	0.2	0.1	0.2544	0.0773	0.2481	0.0887	0.1148
		0.4	0.1678	0.2795	0.1508	0.1884	0.2230
		0.8	0.3921	0.3457	0.3876	0.3474	0.4105
	0.4	0.1	0.1558	0.1237	0.0576	0.1429	0.1632
		0.4	0.1583	0.1271	0.1860	0.1948	0.2238
		0.8	0.4045	0.5474	0.2710	0.4405	0.2535
10,000	0.2	0.1	0.2571	0.1099	0.1134	0.1201	0.1193
		0.4	0.2043	0.1863	0.1444	0.1446	0.1767
		0.8	0.3508	0.5678	0.4920	0.4279	0.4999
	0.4	0.1	0.1624	0.1086	0.1242	0.1544	0.1603
		0.4	0.1619	0.1266	0.1887	0.1977	0.2009
		0.8	0.5450	0.6937	0.4035	0.5953	0.6834

Shaded cells indicate IBS < 0.25.

**Table 12.** Example Survival-LCS Rule-Set: This Table Summarizes the Top 10 Rules (Sorted by Numerosity) of a Survival LCS Model Trained on the Same Simulated Dataset with an Epistatic Model and Gamma Baseline Distribution Also Used in Figure 7

Specified Feature Names	Specified Values	Event Interval	Accuracy	Numerosity	Iteration Initialized	Specificity	Deletion Probability	Correct Count Match	Match Count
M0P5, M0P6	0, 1	[01.23–49.27]	0.927	10	95,585	0.02	0.006	18,797	19,068
M0P5, M0P6	2, 1	[38.62–79.00]	0.970	8	111,404	0.02	0.005	6,252	6,370
M0P5, M0P6	2, 2	[50.05–84.20]	0.987	8	111,715	0.02	0.003	1,672	1,673
M0P5, M0P6	0, 0	[39.59–88.35]	0.968	8	148,889	0.02	0.004	5,635	5,837
M0P5, M0P6	2, 1	[22.28–79.00]	0.930	8	162,453	0.02	0.005	2,725	2,732
M0P5, M0P6	0, 1	[02.52–54.03]	0.945	8	180,561	0.02	0.004	3,653	3,661
M0P5, M0P6	0, 1	[02.52–49.27]	0.938	7	95,260	0.02	0.004	18,857	19,129
M0P5, M0P6	2, 1	[26.42–79.00]	0.954	7	155,734	0.02	0.004	3,211	3,218
M0P5, M0P6	0, 0	[36.37–82.34]	0.961	7	187,000	0.02	0.004	1,486	1,546
M0P5	1	[20.46–49.27]	0.964	7	168,582	0.01	0.003	13,869	15,746

A rule’s condition is comprised of specified feature names, and associated specified feature values (i.e., states), while a rule’s action is the “event interval,” and other columns capture a variety of other key rule parameters that define the quality and other characteristics of individual rules.

## Box

CCS Concepts: • **Computing methodologies** → **Rule learning; Genetic algorithms**; • **Applied computing** → Health informatics;
