# Supplementary material for: Survival-LCS: Rule-Based Survival Analysis without Proportional Hazard Assumptions
Source: ACM Trans Evol Learn Optim. Author manuscript; Available in PMC 2026 Sep 2. (PMC13532393; doi:10.1145/3772719)
Supplement: Supplementary file 1 [file NIHMS2205420-supplement-Supplementary_Material.pdf]

Supplemental Materials

Survival-LCS: Rule-Based Survival Analysis Without Proportional Hazard Assumptions

Alexa A. Woodward*, Harsh Bandhey*, Jason H. Moore, and Ryan J. Urbanowicz†

Table of Contents

S1. Methods 2
- S1.1 Survival-LCS Algorithm 2
- S1.2 Simulated Survival Datasets 4
  - S1.2.1 Genetic Models 4
  - S1.2.2 Baseline Hazard Distributions 5
- S1.3 Evaluation Metrics 6
  - S1.3.1 Performance: Integrated Brier Score 6
  - S1.3.2 Statistical Significance: Wilcoxon Rank Sum Test 6
- S1.4 Summary of Analytical Design 7

S2. Results and Discussion 7
- S2.1 Integrated Brier Scores for Performance Analyses Across Different Baselines 7
  - S2.1.1 Random Spline Baseline Distribution (Initial Woodward et al) 7
  - S2.1.2 Random Spline Baseline Distribution 12
  - S2.1.3 Gamma Baseline Distribution 16
  - S2.1.4 Gaussian Baseline Distribution 20
  - S2.1.5 Weibull Baseline Distribution 24
- S2.2 Wilcoxon Rank Sum Tests for Statistical Significance of Performance Differences 28
  - S2.2.1 Stratification by Genetic Models 28
  - S2.2.2 Stratification by Number of Features 30
  - S2.2.3 Stratification by Censoring 32
  - S2.2.4 Stratification by Minor Allele Frequency 34
- S2.3 Supplemental Discussion of Findings 36
  - S2.3.1 Robustness to Violations of Proportional Hazards 36
  - S2.3.2 Efficiency in Simple Scenarios 36
  - S2.3.3 Feature Dimensionality 36
  - S2.3.4. Minor Allele Frequency 37
  - S2.3.5 Consistency Across Baseline Hazard Distributions 37

S1. Methods

To investigate how Survival-LCS behaves across a wide variety of survival data conditions, we designed a series of simulation experiments that captured different genetic structures and survival processes. The goal of these simulations was not only to measure predictive accuracy but also to explore robustness in cases where proportional hazard assumptions are violated and to examine sensitivity to data features such as high dimensionality, right-censoring, and allele frequency distributions.

S1.1 Survival-LCS Algorithm

Survival-LCS is a **rule-based survival modeling framework** that learns an ensemble of *IF:THEN* rules that maps subgroups of instances to survival time ranges. This rule-based approach enables Survival-LCS to capture **nonlinear effects, gene-gene interactions, and heterogeneous subpopulations**, while remaining interpretable (i.e., each rule interpretably maps feature value states to survival outcomes). All hyperparameter values available for the Survival-LCS algorithm are documented in **Table S1**, along with the hyperparameter value settings used in this study. The majority of these hyperparameters are the same default settings used by many LCS algorithms. The only two key Survival-LCS hyperparameters that are expected to strongly impact algorithm performance in this study is `learning iterations' and `N' maximum population size

Survival-LCS Parameter	Value	Notes
`learning_iterations`	**200000**	Override from Woodward et al. (default 50000)
`N` (maximum population size)	**2000**	Override from Woodward et al. (default 1000)
`nu`	1	Power on accuracy
`chi`	0.8	GA crossover prob.
`mu`	0.04	GA mutation prob.
`upsilon`	0.04	Mutation/adjustment rate
`theta_GA`	25	GA trigger threshold
`theta_del`	20	Deletion experience threshold
`theta_sub`	20	Subsumption experience threshold

acc_sub	0.99	Accuracy requirement for subsumption
beta	0.2	Learning rate for averages
delta	0.1	Deletion parameter
init_fitness	0.01	Initial classifier fitness
fitness_reduction	0.1	Fitness reduction on GA offspring
theta_sel	0.5	Fraction in tournament selection
rule_specificity_limit	None	If not None, caps specificity
do_correct_set_subsumption	False	Subsumption in [C] is disabled
do_GA_subsumption	True	Subsumption between offspring/parents
selection_method	"tournament"	GA parent selection
do_attribute_tracking	True	Enable AT
do_attribute_feedback	True	Enable AF
attribute_tracking_method	"add"	AT update rule
attribute_tracking_beta	0.1	AT learning rate
expert_knowledge	None	EK unused
rule_compaction	"QRF"	Rule compaction method
reboot_filename	None	Reboot disabled
discrete_attribute_limit	10	Threshold for discrete vs continuous
specified_attributes	np.array([])	No forced continuous/discrete
track_accuracy_while_fit	True	Accuracy tracked during training
random_state	**42**	Matches pipeline seed

Table S1. Survival-LCS hyperparameters and default settings used in this study.

S1.2 Simulated Survival Datasets

S1.2.1 Genetic Models

We simulated outcomes under four genetic architectures (i.e. models): **additive, epistatic, heterogeneous, and univariate**. These scenarios cover a range of relationships seen in survival data, from simple single-feature effects to complex interactions and instance-subgroup-specific hazards. The names of the predictive features for each model in the scenario where the minor allele frequency (MAF) is either 0.2 or 0.4 are listed in **Table S2**. Of note, all underlying genetic models simulated using GAMETES were generated to have a heritability of 0.2 (creating very noisy survival data simulations). We expect that the additive model would have the strongest signal of all of these simulated datasets since we are additively combining the 0.2 heritability effect across three simulated predictive features. In contrast, we expect the heterogeneous dataset to have the weakest signal due to the underlying models only being relevant in a given half of all simulated dataset instances.

- **Additive:** Outcomes are explained by features that each make an independent, proportional contribution to the hazard. *Example:* M0P1 and M1P3 both raise risk, and their effects add together without interaction.
- **Epistatic:** Outcomes depend entirely on interactions. Individual features have no effect alone, but pairs (e.g., M0P3 x M0P4) become predictive when considered jointly.
- **Heterogeneous:** Features define subgroups with distinct hazard patterns, producing non-monotonic effects. In these simulations M0P2 had a univariate effect modeled in one half of the data instances, while the other two features had a two-way epistatic interaction that was modeled in a separate half of the instances. Overall this dilutes the signal of either heterogeneous pattern within the dataset as a whole.
- **Univariate:** Outcomes are driven by a single univariate feature, serving as a simple baseline case (e.g., M0P1).

**Genetic model**	**MAF = 0.2**	**MAF = 0.4**
Additive	M0P1, M1P3, M1P4	M0P5, M0P6, M1P2
Epistatic	M0P3, M0P4	M0P5, M0P6
Heterogeneous	M0P2, M1P3, M1P4	M0P2, M1P5, M1P6
Univariate	M0P1	M0P2

Table S2. Predictive features used across simulated dataset scenarios (i.e. genetic model and MAF).

Simulated dataset scenarios were further varied by **censoring proportions**, **total feature counts, and the baseline hazard distribution**. Together, these variations created a rich simulation study that systematically probed how genetic architecture, feature dimensionality, allele distributions, and censoring interact to influence survival modeling outcomes. The full simulation design is summarized in **Table S3**.

**Dataset Property**	**Parameters**	**Descriptions**
Genetic models	`me` (univariate), `epi` (epistatic), `het` (heterogeneous), `add` (additive)	Simulation architectures
Feature counts	`f100`, `f1000`, `f10000`	Number of simulated features
Minor allele frequencies	`maf0.2`, `maf0.4`	Simulated allele frequencies
Censoring proportions	`0.1`, `0.4`, `0.8`	Levels of censoring applied
Time horizon	`T = 100`	Maximum follow-up time

Table S3. Dataset properties and simulation parameters.

S1.2.2 Baseline Hazard Distributions

To ensure generalizability across survival settings, we stratified and tested genetic models across multiple baseline hazard distributions, ranging from flexible splines to classical parametric forms:

- **Random Spline (Initial Woodward et al. Analysis)**: Flexible spline-based hazards with random initialization from a previous publication.
- **Random Spline**: A separate initialization of the spline-based hazard, used to confirm robustness (used as the primary benchmark).
- **Gamma Distribution**: A skewed parametric form capturing asymmetric event times.
- **Gaussian Distribution**: It has a symmetric hazard structure with smooth failure times, which mimics many real-life distributions.
- **Weibull Distribution**: A classical parametric family widely used in survival analysis due to its monotonic hazard baseline.

This setup allowed us to test performance across both monotonic and non-monotonic survival structures. The hazard types and their parameterizations are listed in Table S4.

Baseline Hazard Distributions	Parameters	Description
Random spline (initial)	`pmethod="random", knots=8`	Flexible monotonic spline
Random spline	`pmethod="random", knots=8`	Flexible monotonic spline
Gamma	Shape = **2**, Scale = **0.1**	Parametric skewed distribution
Gaussian	Mean = **0.5**, SD = **0.1**	Symmetric hazard distribution
Weibull	Shape = **0.63**, Scale = **0.33**	Classical monotonic hazard

Table S4. Baseline hazard distributions considered in simulation experiments.

S1.3 Evaluation Metrics

S1.3.1 Performance: Integrated Brier Score

Performance was measured using the **Integrated Brier Score (IBS)**, which summarizes prediction error across the full follow-up period. At any given time point *t*, the Brier Score is the squared difference between the predicted survival probability and the observed event indicator. Integrating across time produces the IBS, a comprehensive measure of both calibration and discrimination.

A **lower IBS (= < 0.25)** indicates accurate predictions across time, and **a higher IBS value greater than 0.25 indicates a** poor model fit. All results in Section S2.1 (Tables S6 to S25) report IBS values with confidence intervals calculated by cross-validation.

S1.3.2 Statistical Significance: Wilcoxon Rank Sum Test

To formally evaluate whether observed differences were meaningful, we applied the Wilcoxon rank sum test to IBS values across simulation replicates. Unlike parametric tests, this approach does not assume normality and is more appropriate for performance comparisons in high-variance settings. Significance was assessed at $p < 0.05$. Stratified analyses were carried out across:

1. **Genetic Model** (additive, epistatic, heterogeneous, univariate)
2. **Number of Features** (100, 1000, 10000)

3. **Censoring Proportion** (0.1, 0.4, 0.8)
4. **Minor Allele Frequency** (0.2, 0.4)

Results of these tests are presented in Section S2.2 (Tables S26 to S45).

S1.4 Summary of Analytical Design

In total, our supplementary analyses span **four baseline hazard distributions, four genetic architectures (i.e. models), three different total feature counts, three different censoring proportions, and allele frequencies across different cross-validation** folds. This design ensures that conclusions about Survival-LCS are not artifacts of a single assumption but reflect performance across a wide spectrum of survival modeling challenges.

S2. Results and Discussion

S2.1 Integrated Brier Scores for Performance Analyses Across Different Baselines

The following tables present IBS results for Survival-LCS and comparator methods across the different hazard distributions. Lower IBS values correspond to stronger calibration and discrimination.

S2.1.1 Random Spline Baseline Distribution (Initial Woodward et al)

These tables (S6-S9) display IBS results using the originally defined random spinal baseline distribution with only 50,000 iterations and a rule population size of 1000 from the initial Woodward et al. paper as a point of comparison.

Features	MAF	Censoring	IBS	Lower CI	Upper CI
100	0.2	10%	0.05596	0.04367	0.07135
		40%	0.17522	0.11566	0.24583
		80%	0.12323	-0.00505	0.28295
	0.4	10%	0.15227	0.11503	0.19641
		40%	0.12062	0.08803	0.15947
		80%	0.21982	0.06747	0.40044
1000	0.2	10%	0.07787	0.05982	0.09931
		40%	0.20688	0.1418	0.28404
		80%	0.3978	0.21096	0.61956
	0.4	10%	0.12946	0.11142	0.15146
		40%	0.14348	0.08292	0.21531
		80%	0.43387	0.03364	0.90956
10000	0.2	10%	0.04087	0.02907	0.05522
		40%	0.17304	0.11839	0.23955
		80%	0.40003	0.26204	0.56428
	0.4	10%	0.1058	0.06827	0.1503
		40%	0.09626	0.06432	0.13423
		80%	0.08435	0.01968	0.16197

Table S6. Integrated Brier Scores for additive genetic effects under random spline baseline distribution (Initial Woodward et al).

Features	MAF	Censoring	IBS	Lower CI	Upper CI
100	0.2	10%	0.07328	0.0401	0.11281
		40%	0.20109	0.14916	0.26563
		80%	0.30844	0.13179	0.521
	0.4	10%	0.15533	0.13632	0.17838
		40%	0.28311	0.21687	0.36185
		80%	0.17654	0.06272	0.31162
1000	0.2	10%	0.23361	0.21296	0.25863
		40%	0.14203	0.07883	0.21714
		80%	0.18466	0.03748	0.35911
	0.4	10%	0.08712	0.05733	0.12257
		40%	0.14956	0.11119	0.19519
		80%	0.4093	0.27758	0.57186
10000	0.2	10%	0.07273	0.0587	0.09003
		40%	0.13399	0.06399	0.21741
		80%	0.14903	0.05822	0.26051
	0.4	10%	0.10553	0.07107	0.14729
		40%	0.24696	0.17188	0.33598
		80%	0.55478	0.37764	0.76722

Table S7. Integrated Brier Scores for epistatic genetic models under random spline baseline distribution (Initial Woodward et al).

Features	MAF	Censoring	IBS	Lower CI	Upper CI
100	0.2	10%	0.14149	0.10645	0.18304
		40%	0.18536	0.14444	0.23403
		80%	0.36859	0.20219	0.56586
	0.4	10%	0.09294	0.05655	0.13616
		40%	0.13661	0.09028	0.19196
		80%	0.81028	0.45764	1.26555
1000	0.2	10%	0.28285	0.21959	0.35785
		40%	0.13791	0.10381	0.17931
		80%	0.27066	0.09739	0.48117
	0.4	10%	0.14966	0.12946	0.17361
		40%	0.13822	0.10062	0.18281
		80%	0.34466	0.15796	0.57242
10000	0.2	10%	0.11434	0.09005	0.14384
		40%	0.29555	0.23873	0.36295
		80%	0.45371	0.45371	0.45371
	0.4	10%	0.12263	0.08236	0.17141
		40%	0.23196	0.18946	0.28235
		80%	0.20232	0.02772	0.4112

Table S8. Integrated Brier Scores for heterogeneous effect distributions under random spline baseline distribution (Initial Woodward et al).

Features	MAF	Censoring	IBS	Lower CI	Upper CI
100	0.2	10%	0.15869	0.12598	0.19834
		40%	0.17971	0.1351	0.23396
		80%	0.38818	0.13684	0.69481
	0.4	10%	0.15727	0.03398	0.30658
		40%	0.17812	0.02345	0.36248
		80%	0.2407	0.06728	0.44679
1000	0.2	10%	0.25443	0.21735	0.29943
		40%	0.16783	0.07827	0.27504
		80%	0.39212	0.29666	0.50789
	0.4	10%	0.1558	0.12155	0.19732
		40%	0.15825	0.08602	0.24688
		80%	0.40453	0.08995	0.77749
10000	0.2	10%	0.25706	0.22204	0.29959
		40%	0.20427	0.1623	0.25512
		80%	0.35077	0.19473	0.53575
	0.4	10%	0.16244	0.10666	0.2319
		40%	0.16191	0.12742	0.20448
		80%	0.54501	0.25259	0.90045

Table S9. Integrated Brier Scores for univariate datasets under random spline baseline distribution (Initial Woodward et al).

S2.1.2 Random Spline Baseline Distribution

These tables (S10 - S13) report results for a new initialization of the random spline baseline distribution to validate and run with 200,000 iterations of survival-LCS and a rule population size of 2000 rules, serving as the primary benchmark of model performance.

Features	MAF	Censoring	IBS	Lower CI	Upper CI
100	0.2	0.1	0.078015	0.042674	0.114603
		0.4	0.164714	0.090058	0.239369
		0.8	0.190226	0.055990	0.324461
	0.4	0.1	0.088803	0.069796	0.112968
		0.4	0.172076	0.090961	0.272843
		0.8	0.329178	0.081326	0.589475
1000	0.2	0.1	0.114779	0.102001	0.127557
		0.4	0.097106	0.053444	0.146084
		0.8	0.348224	0.244555	0.454068
	0.4	0.1	0.102648	0.071486	0.147299
		0.4	0.124991	0.098373	0.160810
		0.8	0.368877	0.096111	0.655979
10000	0.2	0.1	0.064559	0.048235	0.087967
		0.4	0.167249	0.127871	0.206626
		0.8	0.369870	0.230680	0.571573
	0.4	0.1	0.092319	0.066903	0.117735
		0.4	0.211989	0.112643	0.313431
		0.8	0.460139	-0.028811	0.952894

Table S10. Integrated Brier Scores for additive datasets under a new initialization of random spline baseline distribution.

Features	MAF	Censoring	IBS	Lower CI	Upper CI
100	0.2	0.1	0.126381	0.060642	0.225259
		0.4	0.115775	0.117325	0.198442
		0.8	0.133137	0.054068	0.210693
	0.4	0.1	0.102788	0.098570	0.200051
		0.4	0.164183	0.114857	0.213439
		0.8	0.267425	0.161167	0.373684
1000	0.2	0.1	0.143653	0.085228	0.202078
		0.4	0.181936	0.152604	0.245334
		0.8	0.355909	0.058084	0.649053
	0.4	0.1	0.130846	0.103575	0.168429
		0.4	0.160033	0.096248	0.230378
		0.8	0.197249	0.076292	0.320053
10000	0.2	0.1	0.159641	0.094176	0.225106
		0.4	0.176524	0.067541	0.301250
		0.8	0.412843	0.221456	0.605500
	0.4	0.1	0.187950	0.148098	0.227802
		0.4	0.117467	0.071896	0.163039
		0.8	0.072181	0.023427	0.113182

Table S11. Integrated Brier Scores for epistatic datasets under a new initialization of random spline baseline distribution.

Features	MAF	Censoring	IBS	Lower CI	Upper CI
100	0.2	0.1	0.179879	0.146343	0.215607
		0.4	0.122803	0.158620	0.205961
		0.8	0.227514	-0.048290	0.649600
	0.4	0.1	0.161742	0.119394	0.204091
		0.4	0.200740	0.155222	0.271456
		0.8	0.369916	0.180590	0.558554
1000	0.2	0.1	0.113107	0.088214	0.144726
		0.4	0.148436	0.104308	0.193833
		0.8	0.252511	0.090545	0.352026
	0.4	0.1	0.101088	0.059532	0.144600
		0.4	0.155569	0.091543	0.224663
		0.8	0.207466	0.056257	0.330981
10000	0.2	0.1	0.171432	0.157956	0.188239
		0.4	0.134688	0.075176	0.202035
		0.8	0.102993	0.054766	0.112063
	0.4	0.1	0.241818	0.166723	0.320564
		0.4	0.202188	0.133691	0.270685
		0.8	0.306650	0.014625	0.600351

Table S12. Integrated Brier Scores for heterogeneous datasets under a new initialization of random spline baseline distribution.

Features	MAF	Censoring	IBS	Lower CI	Upper CI
100	0.2	0.1	0.129865	0.095666	0.175660
		0.4	0.160364	0.073138	0.255526
		0.8	0.342781	0.017553	0.666432
	0.4	0.1	0.137622	0.088541	0.201041
		0.4	0.166774	0.015136	0.318433
		0.8	0.318525	0.016062	0.632657
1000	0.2	0.1	0.077318	0.045053	0.118129
		0.4	0.279521	0.202187	0.356855
		0.8	0.345707	-0.059261	0.521402
	0.4	0.1	0.123749	0.077613	0.176296
		0.4	0.127102	-0.014465	0.269610
		0.8	0.547366	0.181111	0.909876
10000	0.2	0.1	0.109922	0.073209	0.146635
		0.4	0.186272	0.111492	0.261051
		0.8	0.567774	0.177701	0.920792
	0.4	0.1	0.108604	0.066978	0.151517
		0.4	0.126600	0.073465	0.231637
		0.8	0.693660	0.508127	0.909130

Table S13. Integrated Brier Scores for univariate datasets under a new initialization of random spline baseline distribution.

S2.1.3 Gamma Baseline Distribution

Tables S14 to S17 present results under a Gamma baseline distribution, capturing skewed event-time patterns.

Features	MAF	Censoring	IBS	Lower CI	Upper CI
100	0.2	0.1	0.103528	0.084338	0.138417
		0.4	0.119194	0.046101	0.205471
		0.8	0.437465	0.128134	0.841290
	0.4	0.1	0.117622	0.076516	0.199552
		0.4	0.145416	0.087669	0.203163
		0.8	0.502305	0.154607	0.846506
1000	0.2	0.1	0.054673	0.033614	0.075733
		0.4	0.110652	0.063090	0.159328
		0.8	0.395117	0.100811	0.689423
	0.4	0.1	0.042369	0.006730	0.083100
		0.4	0.282104	0.214483	0.353816
		0.8	0.172292	0.091017	0.249737
10000	0.2	0.1	0.123008	0.096931	0.154051
		0.4	0.365853	0.309867	0.446753
		0.8	0.389843	0.156279	0.623407
	0.4	0.1	0.099293	0.073864	0.130127
		0.4	0.084492	0.049797	0.115991
		0.8	0.478710	0.182300	0.791665

Table S14. Integrated Brier Scores for additive datasets under the Gamma baseline distribution.

Features	MAF	Censoring	IBS	Lower CI	Upper CI
100	0.2	0.1	0.113787	0.044126	0.185577
		0.4	0.125417	0.083179	0.168976
		0.8	0.259140	-0.017292	0.565919
	0.4	0.1	0.128511	0.079180	0.187003
		0.4	0.179122	0.117751	0.241516
		0.8	0.172943	0.073835	0.338107
1000	0.2	0.1	0.083578	0.059221	0.115004
		0.4	0.127285	0.065173	0.185227
		0.8	0.357790	0.063776	0.653942
	0.4	0.1	0.188346	0.144916	0.260257
		0.4	0.282043	0.168720	0.399898
		0.8	0.461732	0.028359	0.890859
10000	0.2	0.1	0.066359	0.043637	0.093182
		0.4	0.215187	0.177630	0.252744
		0.8	0.110685	0.034576	0.183132
	0.4	0.1	0.129119	0.102477	0.158272
		0.4	0.243902	0.181687	0.306117
		0.8	0.357084	0.261467	0.448702

Table S15. Integrated Brier Scores for epistatic datasets under the Gamma baseline distribution.

Features	MAF	Censoring	IBS	Lower CI	Upper CI
100	0.2	0.1	0.248439	0.019742	0.477136
		0.4	0.118526	0.076079	0.213703
		0.8	1.200026	-0.181861	2.432343
	0.4	0.1	0.144933	0.127820	0.174418
		0.4	0.226637	0.142959	0.313920
		0.8	0.345881	0.083564	0.605345
1000	0.2	0.1	0.109210	0.093817	0.162048
		0.4	0.159016	0.108241	0.215673
		0.8	0.233496	0.090146	0.376846
	0.4	0.1	0.169349	0.087478	0.254710
		0.4	0.219589	0.202309	0.236869
		0.8	0.175871	0.122376	0.226250
10000	0.2	0.1	0.060879	0.035103	0.094605
		0.4	0.210086	0.124322	0.300995
		0.8	0.408474	0.009490	0.807459
	0.4	0.1	0.067755	0.037439	0.099530
		0.4	0.254714	0.084103	0.425326
		0.8	0.204176	0.150849	0.424771

Table S16. Integrated Brier Scores for heterogeneous datasets under the Gamma baseline distribution.

Features	MAF	Censoring	IBS	Lower CI	Upper CI
100	0.2	0.1	0.264533	0.225389	0.305097
		0.4	0.342638	0.283773	0.404366
		0.8	0.442103	-0.005565	0.889017
	0.4	0.1	0.134391	0.106015	0.200561
		0.4	0.167229	0.117876	0.252517
		0.8	0.276712	0.005685	0.559655
1000	0.2	0.1	0.248090	0.220023	0.284009
		0.4	0.150836	0.029357	0.280949
		0.8	0.387589	0.061500	0.712598
	0.4	0.1	0.057635	0.025319	0.093743
		0.4	0.186009	0.017956	0.354061
		0.8	0.270952	-0.022964	0.564868
10000	0.2	0.1	0.113387	0.017604	0.221462
		0.4	0.144386	0.068062	0.222662
		0.8	0.491963	0.321960	0.661562
	0.4	0.1	0.124227	0.040589	0.207866
		0.4	0.188673	0.100727	0.282210
		0.8	0.403517	0.189329	0.619697

Table S17. Integrated Brier Scores for univariate datasets under the Gamma baseline distribution.

S2.1.4 Gaussian Baseline Distribution

Tables S18 to S21 show results under a symmetric Gaussian hazard distribution.

Features	MAF	Censoring	IBS	Lower CI	Upper CI
100	0.2	0.1	0.102010	0.055909	0.149743
		0.4	0.111383	0.039103	0.185238
		0.8	0.362891	0.116309	0.609473
	0.4	0.1	0.162187	0.135679	0.189791
		0.4	0.225228	0.143511	0.313546
		0.8	0.339510	0.254950	0.461795
1000	0.2	0.1	0.114182	0.085928	0.162847
		0.4	0.171302	0.083705	0.258900
		0.8	0.283454	0.035559	0.555483
	0.4	0.1	0.130724	0.105426	0.165612
		0.4	0.110979	0.052659	0.210787
		0.8	0.513697	0.322023	0.728868
10000	0.2	0.1	0.077548	0.048018	0.111047
		0.4	0.176852	0.098352	0.263030
		0.8	0.537204	0.122477	0.913948
	0.4	0.1	0.116271	0.096787	0.138102
		0.4	0.200316	0.117761	0.286130
		0.8	0.414046	0.143494	0.713014

Table S18. Integrated Brier Scores for additive datasets under a Gaussian baseline distribution.

Features	MAF	Censoring	IBS	Lower CI	Upper CI
100	0.2	0.1	0.120828	0.102501	0.139156
		0.4	0.146127	0.102533	0.189721
		0.8	1.146647	-0.745773	3.039067
	0.4	0.1	0.122549	0.084545	0.193093
		0.4	0.123939	0.063191	0.182980
		0.8	0.406985	0.085041	0.742162
1000	0.2	0.1	0.117091	0.103503	0.136518
		0.4	0.210886	0.137526	0.284246
		0.8	0.437347	-0.105031	1.071302
	0.4	0.1	0.096488	0.067628	0.142288
		0.4	0.113645	0.079678	0.156371
		0.8	0.174514	0.076803	0.274462
10000	0.2	0.1	0.080011	0.057323	0.102699
		0.4	0.179335	0.116371	0.229944
		0.8	0.454831	-0.164337	1.040194
	0.4	0.1	0.148290	0.089724	0.206855
		0.4	0.126539	0.075715	0.261726
		0.8	0.496331	0.284158	0.713977

Table S19. Integrated Brier Scores for epistatic datasets under a Gaussian baseline distribution.

Features	MAF	Censoring	IBS	Lower CI	Upper CI
100	0.2	0.1	0.191389	0.172324	0.256256
		0.4	0.196620	0.065028	0.330225
		0.8	0.454244	0.234440	0.683610
	0.4	0.1	0.072707	0.056294	0.097628
		0.4	0.210554	0.161083	0.268930
		0.8	0.245738	-0.121510	0.789739
1000	0.2	0.1	0.134847	0.096089	0.173606
		0.4	0.309618	0.171412	0.459190
		0.8	0.468941	0.120806	0.817076
	0.4	0.1	0.148739	0.108497	0.188980
		0.4	0.170038	0.129086	0.213036
		0.8	0.168158	0.048597	0.287719
10000	0.2	0.1	0.207169	0.179718	0.238788
		0.4	0.250049	0.192606	0.312874
		0.8	0.257475	0.107132	0.407818
	0.4	0.1	0.079865	0.071505	0.092848
		0.4	0.176214	0.108704	0.246161
		0.8	0.204212	0.079348	0.329075

Table S20. Integrated Brier Scores for heterogeneous datasets under a Gaussian baseline distribution.

Features	MAF	Censoring	IBS	Lower CI	Upper CI
100	0.2	0.1	0.090411	0.060677	0.122372
		0.4	0.193904	0.137872	0.255016
		0.8	0.338262	0.018577	0.645204
	0.4	0.1	0.098929	0.029897	0.169845
		0.4	0.165447	0.005946	0.327731
		0.8	0.211794	0.005492	0.418096
1000	0.2	0.1	0.088699	0.057707	0.160273
		0.4	0.188365	0.108372	0.268357
		0.8	0.347403	0.246755	0.470511
	0.4	0.1	0.142889	0.093124	0.194066
		0.4	0.194814	0.030684	0.365573
		0.8	0.440508	0.002402	0.878613
10000	0.2	0.1	0.120086	0.084889	0.157339
		0.4	0.144644	0.039370	0.261650
		0.8	0.427947	0.201135	0.675892
	0.4	0.1	0.154411	0.086433	0.225530
		0.4	0.197654	0.012830	0.382479
		0.8	0.595302	0.202824	1.019332

Table S21. Integrated Brier Scores for univariate datasets under a Gaussian baseline distribution.

S2.1.5 Weibull Baseline Distribution

Tables S22 to S25 report findings under the Weibull family, one of the most widely applied parametric survival models.

Features	MAF	Censoring	IBS	Lower CI	Upper CI
100	0.2	0.1	0.043417	0.018134	0.070019
		0.4	0.168107	0.082208	0.226547
		0.8	0.259588	-0.061978	0.569311
	0.4	0.1	0.141799	0.085638	0.207186
		0.4	0.158139	0.075388	0.242668
		0.8	0.088547	0.033974	0.143119
1000	0.2	0.1	0.156694	0.116999	0.200460
		0.4	0.160384	0.042592	0.276348
		0.8	0.278890	0.098469	0.491365
	0.4	0.1	0.184077	0.126643	0.241511
		0.4	0.130272	0.099335	0.193280
		0.8	1.320522	0.038418	0.942169
10000	0.2	0.1	0.089834	0.066343	0.113325
		0.4	0.190845	0.110225	0.285465
		0.8	0.413350	0.101487	0.767039
	0.4	0.1	0.140456	0.102101	0.178811
		0.4	0.173146	0.105467	0.262445
		0.8	0.546365	0.286798	0.804560

Table S22. Integrated Brier Scores for additive datasets under Weibull baseline distribution.

Features	MAF	Censoring	IBS	Lower CI	Upper CI
100	0.2	0.1	0.075296	0.037316	0.116204
		0.4	0.158157	0.118899	0.197415
		0.8	0.099371	0.013016	0.185726
	0.4	0.1	0.196353	0.153027	0.243652
		0.4	0.103887	0.049100	0.158674
		0.8	0.335832	0.237943	0.491668
1000	0.2	0.1	0.083181	0.060657	0.109312
		0.4	0.202285	0.114260	0.293164
		0.8	0.312388	0.094906	0.525809
	0.4	0.1	0.161537	0.147653	0.179784
		0.4	0.137831	0.077380	0.198282
		0.8	0.422626	0.124916	0.718625
10000	0.2	0.1	0.144268	0.127244	0.170564
		0.4	0.140908	0.044149	0.241964
		0.8	0.296842	0.013887	0.579797
	0.4	0.1	0.107156	0.068179	0.146132
		0.4	0.114165	0.077352	0.179558
		0.8	0.342851	0.210905	0.453965

Table S23. Integrated Brier Scores for epistatic datasets under Weibull baseline distribution.

Features	MAF	Censoring	IBS	Lower CI	Upper CI
100	0.2	0.1	0.082066	0.064074	0.104531
		0.4	0.096593	0.059341	0.134468
		0.8	0.204689	0.077464	0.331915
	0.4	0.1	0.097643	0.063980	0.133259
		0.4	0.109922	0.048985	0.177179
		0.8	0.516673	0.235385	0.818289
1000	0.2	0.1	0.194260	0.178013	0.220385
		0.4	0.156226	0.119905	0.217610
		0.8	0.389599	0.146301	0.636182
	0.4	0.1	0.163452	0.145692	0.181212
		0.4	0.221165	0.147889	0.294441
		0.8	0.429686	0.151461	0.747163
10000	0.2	0.1	0.280300	0.230480	0.348667
		0.4	0.243747	0.147098	0.347505
		0.8	0.353385	0.147447	0.590074
	0.4	0.1	0.088503	0.068533	0.110262
		0.4	0.215597	0.145544	0.285651
		0.8	0.289329	0.103641	0.464939

Table S24. Integrated Brier Scores for heterogeneous datasets under Weibull baseline distribution.

Features	MAF	Censoring	IBS	Lower CI	Upper CI
100	0.2	0.1	0.225066	0.184984	0.265588
		0.4	0.165546	0.019126	0.324419
		0.8	0.276175	0.141909	0.583402
	0.4	0.1	0.097462	0.066364	0.128561
		0.4	0.137168	0.058547	0.236130
		0.8	0.154028	-0.197472	0.505529
1000	0.2	0.1	0.114785	0.085242	0.144327
		0.4	0.223001	0.101780	0.369485
		0.8	0.410480	0.110924	0.725582
	0.4	0.1	0.163235	0.148941	0.177529
		0.4	0.223769	0.030594	0.416943
		0.8	0.253488	0.125862	0.381115
10000	0.2	0.1	0.119271	0.034815	0.208222
		0.4	0.176730	0.046356	0.319282
		0.8	0.499929	0.351990	0.642802
	0.4	0.1	0.160302	0.143783	0.180793
		0.4	0.200950	0.138188	0.266385
		0.8	0.683380	0.275983	1.090777

Table S25. Integrated Brier Scores for univariate datasets under Weibull baseline distribution.

S2.2 Wilcoxon Rank Sum Tests for Statistical Significance of Performance Differences

Wilcoxon rank sum tests evaluate whether the differences observed in IBS are statistically significant.

S2.2.1 Stratification by Genetic Models

Tables S26 to S30 summarize comparisons across additive, epistatic, heterogeneous, and univariate settings.

model 2<br>model 1	add	epi	het	me
**add**	NaN	0.733727	0.048279	0.012032
**epi**	0.733727	NaN	0.264526	0.048279
**het**	0.048279	0.264526	NaN	0.369217
**me**	0.012032	0.048279	0.369217	NaN

Table S26. Wilcoxon tests for random spline baseline distribution (Initial Woodward et al).

model 2<br>model 1	add	epi	het	me
add	NaN	0.966118	0.966118	0.030365
epi	0.966118	NaN	0.324730	0.141518
het	0.966118	0.324730	NaN	0.392738
me	0.030365	0.141518	0.392738	NaN

Table S27. Wilcoxon tests for random spline baseline distribution.

model 2<br>model 1	add	epi	het	me
add	NaN	0.766029	0.898575	0.246208
epi	0.766029	NaN	0.670525	0.264526
het	0.898575	0.670525	NaN	0.579842
me	0.246208	0.264526	0.579842	NaN

Table S28. Wilcoxon tests for a Gamma baseline distribution.

model 2	add	epi	het	me
model 1				
add	NaN	0.670525	0.932281	0.932281
epi	0.670525	NaN	0.865044	0.670525
het	0.932281	0.865044	NaN	0.579842
me	0.932281	0.670525	0.579842	NaN

Table S29. Wilcoxon tests for a Gaussian baseline distribution.

model 2	add	epi	het	me
model 1				
add	NaN	0.212143	0.831726	0.141518
epi	0.212143	NaN	0.043167	0.089767
het	0.831726	0.043167	NaN	0.670525
me	0.141518	0.089767	0.670525	NaN

Table S30. Wilcoxon tests for Weibull baseline distribution.

S2.2.2 Stratification by Number of Features

Tables S31 to S35 show tests stratified by dataset dimensionality. When feature dimensionality increases, comparator models tend to overfit or underperform, reflected in higher IBS and thus significant Wilcoxon results. Survival-LCS maintains stability, demonstrating scalability to multivariate feature sets.

nfeat 2<br>nfeat 1	f100	f1000	f10000
**f100**	NaN	0.473166	0.988813
**f1000**	0.473166	NaN	0.704756
**f10000**	0.988813	0.704756	NaN

Table S31. Wilcoxon tests for random spline baseline distribution (Initial Woodward et al).

nfeat 2<br>nfeat 1	f100	f1000	f10000
f100	NaN	0.921809	0.207617
f1000	0.921809	NaN	0.128027
f10000	0.207617	0.128027	NaN

Table S32. Wilcoxon tests for random spline baseline distribution.

nfeat 2<br>nfeat 1	f100	f1000	f10000
f100	NaN	0.252249	0.527059
f1000	0.252249	NaN	0.564589
f10000	0.527059	0.564589	NaN

Table S33. Wilcoxon tests for a Gamma baseline distribution.

nfeat 2	f100	f1000	f10000
nfeat 1			
f100	NaN	0.899570	0.330505
f1000	0.899570	NaN	0.473166
f10000	0.330505	0.473166	NaN

Table S34. Wilcoxon tests for a Gaussian baseline distribution.

nfeat 2	f100	f1000	f10000
nfeat 1			
f100	NaN	0.001400	0.009576
f1000	0.001400	NaN	0.623094
f10000	0.009576	0.623094	NaN

Table S35. Wilcoxon tests for Weibull baseline distribution.

S2.2.3 Stratification by Censoring

Tables S36 to S40 show tests stratified by proportion of censored observations, to assess performance against right-censoring. Wilcoxon results show that censoring significantly affects performance across all methods, including survival-LCS.

cens 2	cens0.1	cens0.4	cens0.8
**cens 1**			
**cens0.1**	NaN	0.042489	0.000003
**cens0.4**	0.042489	NaN	0.000025
**cens0.8**	0.000003	0.000025	NaN

Table S36. Wilcoxon tests under random spline baseline distribution (Initial Woodward et al).

cens 2	cens0.1	cens0.4	cens0.8
**cens 1**			
cens0.1	NaN	0.013779	0.000010
cens0.4	0.013779	NaN	0.000003
cens0.8	0.000010	0.000003	NaN

Table S37. Wilcoxon tests under a random spline baseline distribution.

cens 2	cens0.1	cens0.4	cens0.8
**cens 1**			
cens0.1	NaN	0.000650	1.192093e-07
cens0.4	6.498098e-04	NaN	1.270771e-04
cens0.8	1.192093e-07	0.000127	NaN

Table S38. Wilcoxon tests under a Gamma baseline distribution.

cens 2	cens0.1	cens0.4	cens0.8
**cens 1**			
cens0.1	NaN	8.344650e-06	1.192093e-07
cens0.4	8.344650e-06	NaN	2.384186e-07
cens0.8	1.192093e-07	2.384186e-07	NaN

Table S39. Wilcoxon tests under a Gaussian baseline distribution.

cens 2	cens0.1	cens0.4	cens0.8
**cens 1**			
cens0.1	NaN	0.031482	5.960464e-07
cens0.4	3.148162e-02	NaN	2.264977e-06
cens0.8	5.960464e-07	0.000002	NaN

Table S40. Wilcoxon tests under the Weibull baseline distribution.

S2.2.4 Stratification by Minor Allele Frequency

Tables S41 to S45 evaluate performance differences under different allele frequencies to evaluate model sensitivity to rare versus common variants. Rare variants pose challenges due to sparse representation. Survival-LCS demonstrates robustness to both common and rare variant settings, with no significant p-value in Wilcoxon tests. This suggests its rules-based structure generalizes well in limited sample sizes for rare alleles.

maf 2	maf0.2	maf0.4
**maf 1**		
**maf0.2**	NaN	0.981432
**maf0.4**	0.981432	NaN

Table S41. Wilcoxon tests under random spline baseline distribution (Initial Woodward et al).

maf 2	maf0.2	maf0.4
**maf 1**		
maf0.2	NaN	0.143293
maf0.4	0.143293	NaN

Table S42. Wilcoxon tests under a random spline baseline distribution.

maf 2	maf0.2	maf0.4
**maf 1**		
maf0.2	NaN	0.726879
maf0.4	0.726879	NaN

Table S43. Wilcoxon tests under a Gamma baseline distribution.

maf 2	maf0.2	maf0.4
**maf 1**		
maf0.2	NaN	0.232497
maf0.4	0.232497	NaN

Table S44. Wilcoxon tests under a Gaussian baseline distribution.

maf 2	maf0.2	maf0.4
**maf 1**		
maf0.2	NaN	0.396375
maf0.4	0.396375	NaN

Table S45. Wilcoxon tests under the Weibull baseline distribution.

S2.3 Supplemental Discussion of Findings

The supplemental results provide an evaluation of Survival-LCS across simulated genetic models, varying feature counts, censoring levels, allele frequencies, and baseline hazard distributions. Here are some findings that show both the strengths and limitations of it:

S2.3.1 Robustness to Violations of Proportional Hazards

Both **epistasis** and **heterogeneity** introduce non-constant effects that violate the proportional hazards (PH) assumption. The clearest strength of Survival-LCS emerges in these datasets, where traditional Cox regression is invalidated. Integrated Brier Scores (IBS) consistently outperform Cox regression under these conditions.

Statistical validation through Wilcoxon rank-sum tests (Tables S26-S30) confirms that these improvements are significant. This demonstrates the ability of a **rule-based framework** to capture nonlinear interactions and subgroup effects that linear hazard structures cannot represent.

S2.3.2 Efficiency in Simple Scenarios

In **additive** and **univariate** settings, where Cox regression assumptions are valid, Survival-LCS performs comparably, though with some efficiency loss. IBS values are often slightly higher than those of Cox regression in these simple scenarios (see Tables S6, S9, S10, S13, S14, S17, S18, and S21).

Wilcoxon results (Tables S26 - S30) indicate that these differences are occasionally, but not consistently, statistically significant. This suggests that while Survival-LCS does not collapse in performance when assumptions favor Cox regression, its **flexibility may come with a small efficiency cost**. Importantly, these losses are minor compared to the substantial gains observed under non-linear or heterogeneous conditions.

S2.3.3 Feature Dimensionality

As the number of simulated features increases (100, 1000, 10,000; see Table S3), Survival-LCS maintains **stable predictive accuracy**. In contrast, Cox regression and parametric models often degrade in performance due to overfitting and instability in high-dimensional settings. Results summarized in Tables S31 - S35 show that Survival-LCS scales well with feature dimensionality, suggesting that its **rule-based ensemble structure generalizes more effectively** in large feature spaces.

S2.3.4. Minor Allele Frequency

Finally, we evaluated the effect of **minor allele frequency (MAF) variation** (see Table S3). No statistically significant differences were observed between low-MAF and high-MAF datasets (Wilcoxon results in Tables S42–S45).

This indicates that Survival-LCS is capable of detecting survival effects even when **causal genetic variants are relatively sparse**, a scenario where proportional hazard models can struggle due to unstable coefficient estimation.

S2.3.5 Consistency Across Baseline Hazard Distributions

Across random spline (initial and new initialization), Gamma, Gaussian, and Weibull baselines, the **relative performance patterns remain consistent** with strong advantages in epistatic and heterogeneous models. This stability across distributions supports the **generalizability of the findings beyond a single hazard assumption**.
